# On the Arrival Time Distribution of Reacting Systems in Ion Mobility Spectrometry

**DOI:** 10.1021/acs.analchem.4c02010

**Published:** 2024-07-15

**Authors:** Alexander Haack, Christoph Schaefer, Stefan Zimmermann

**Affiliations:** Department of Sensors and Measurement Technology, Institute of Electrical Engineering and Measurement Technology, Leibniz University Hannover, 30167 Hannover, Germany

## Abstract

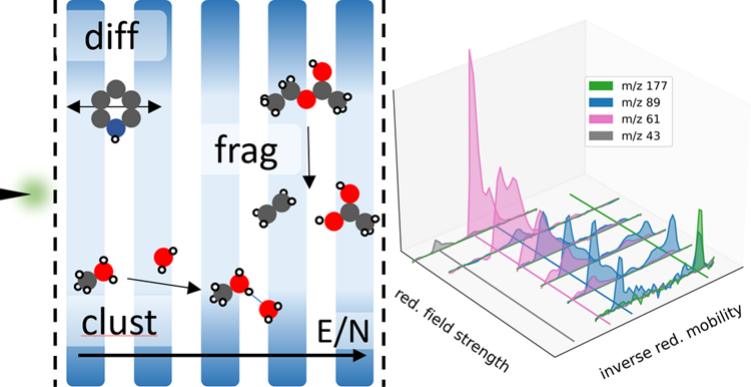

Ion mobility spectrometry (IMS) is a widely used gas-phase separation technique, particularly when coupled with mass spectrometry (MS). Modern IMS instruments often apply elevated reduced field strengths for improved ion separation and ion focusing. These alter the collision dynamics and further drive ion reaction processes that can change the analyte’s structure. As a result, the measured arrival time distribution (ATD) can change with the applied reduced field strengths. In this work, we systematically study how the ion collision dynamics and the ion reaction dynamics, as a function of the reduced field strength, can alter the ATD. To this end, we investigate 2,6-di-*tert*-butylpyridine, methanol, and ethyl acetate using a home-built drift tube IMS coupled to a home-built MS and extensive first-principles Monte Carlo modeling. We show how elevated reduced field strengths can actually lower resolving power through increased ion diffusion and how the field dependency of the ion mobility can introduce uncertainties to collision cross sections (CCS) calculated from the measured mobilities. On top of the collision dynamics, we show how chemical transformation processes that alter the analyte’s CCS, e.g., dynamic clustering or fragmentation, can lead to broadened, shifted, or non-Gaussian ATDs and how sensitive these processes are to the applied field strengths. We highlight how first-principles ion dynamics simulations can help to understand and even harness the mentioned effects.

## Introduction

Ion mobility spectrometry (IMS) has emerged as a powerful separation technique, both as a stand-alone device and when coupled to mass spectrometry (MS). Applications range from trace gas analysis using portable or stand-alone IMS to structural analysis of protein (complexes) when using advanced IMS techniques like trapped IMS (TIMS) or traveling wave IMS (TWIMS) coupled to MS.^1^ The latter have enabled a dramatic increase in resolving power, allowing for the separation of structurally close isomers and probing small differences in the protein structure. To harness the full potential of IMS, in terms of both resolving power and extracting chemical/structural information from the obtained arrival time distributions (ATDs), a fundamental understanding of the dynamic processes occurring during the IMS separation and determining the ATD is necessary. As new-generation IMS devices often operate at significantly elevated reduced electric field strengths, especially field-asymmetric IMS (FAIMS, easily reaching 150 Td) and also TIMS (45–85 Td) and TWIMS (50–160 Td),^1^ there is a growing need to understand ion dynamics outside of the low-field regime. Here, we systematically study ion collision dynamics and ion reaction dynamics over a wide range of reduced field strengths, both experimentally and theoretically. In the following, we will provide a brief review of the fundamental principles that govern the influence of ion dynamics on the ATD observed in the IMS.

### Low Reduced Electric Field Strengths

The movement of analytes in linear separation techniques (chromatography and electrophoresis) may be described by a superposition of a net forward movement (determining the retention/arrival time) and random movement through diffusion (determining the spread of the analyte cloud over time, influencing resolving power). In an idealized drift tube IMS (DTIMS) scenario, the net forward movement can be described by a drift velocity, *v*_*D*_, which is usually expressed as follows^2^
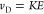
1Here *K* is the ion mobility coefficient and *E* is the electric field strength. Specific to electrophoretic methods, the retention against the electric field and diffusion are both related to collisions with the background medium (solid/liquid/gas). Consequently, the diffusion coefficient, *D*, and the ion mobility coefficient, *K*, are closely related:^2^
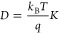
2Here *k*_B_ is the Boltzmann constant, *T* is the absolute temperature, and *q* is the charge of the ion. Moreover, IMS allows us to relate the collision dynamics to the ion’s collision cross section (CCS), a parameter that depends on the size and shape of the analyte ion, as well as the interaction potential between the ion and the collision gas.^3−6^ At low electric fields, this yields^9^
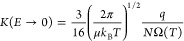
3Here μ is the reduced mass of the ion-neutral pair, *N* is the density of the collision gas, and Ω is the CCS. Note that the collision frequency is directly proportional to the CCS. CCSs can be highly reproducible between laboratories and IMS platforms and can thus be used for molecular identification.^10^ This is a key feature of the IMS: A given CCS determines the arrival time *and* the extent of ion diffusion. As a result, any changes in an ion’s CCS influence both ion mobility and diffusion. Note that eq 3 also shows that differences in the reduced mass can yield different ion mobilities. In fact, as the CCS, too, depends on the reduced mass^3^ and the distribution of the mass in the molecule,^11^ IMS enables the separation of isotopomers.^12,13^

Changes in an ion’s CCS (and mass) can occur through ion transformation processes. The influence of chemical reactions during separation is long known in chromatography and electrophoresis, and two limiting cases are usually identified: (A) The reactions are fast compared to the retention/arrival time and (B) the reactions are in the same order of magnitude or slower than the retention/arrival time. In the first case, peaks are shifted and potentially broadened, but their shape is usually retained. This can be used to determine the equilibrium constants of the ongoing reactions.^14^ In the second case, non-Gaussian peak shapes are observed, which can provide insights into the respective reaction kinetics.^15^ It might also be possible to switch between these two limiting cases, as was demonstrated in affinity chromatography, by altering the flow rate, allowing us to measure both equilibrium constants and rate constants.^16^

In IMS, “fast” reactions are usually observed when two (or more) conformers of the same ion can readily interconvert at a high enough temperature^17,18^ or if dynamic ion–solvent clustering occurs,^19−24^ a fact that is essential for FAIMS when spiked with chemical modifiers.^25−29^ In these situations, it is possible to define an ensemble drift velocity as
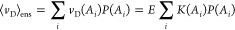
4Here *P*(*A*_*i*_) is the relative population of conformer *A*_*i*_. This can also be understood in terms of ensemble mobility, ⟨*K*⟩_ens_, as represented on the rhs of eq 4. On the other hand, slow or irreversible chemistry was observed in IMS for ion-neutral adduct formation at low neutral concentrations^30−32^ or cold temperatures,^33^ the fragmentation of ions,^34,35^ or conformational dynamics of flexible ions,^17,18,36,37^ all resulting in non-Gaussian peak shapes. Importantly, eq 4 needs to be replaced by a more complex picture. For a bistable system, Poyer et al.^18^ have derived analytical equations for modeling the non-Gaussian ATD in IMS, showing excellent agreement with the experiment and allowing extraction of the interconversion rates.

### High Reduced Electric Field Strengths

In most separation techniques, the net forward velocity of analytes is small compared to thermal velocities on a molecular level. This is not true for IMS when operated at elevated reduced field strengths. Here, the drift velocity can become significant compared to the thermal velocities, distorting the three-dimensional (3D) velocity distribution of the ions in the direction of the field and, to a minor extent, perpendicular to the field.^38^ As a result, the collision dynamics of the ions change significantly, and eqs 2 and 3 become invalid.

Recently, there have been attempts to model the full 3D velocity distribution of polyatomic species as a function of reduced field strength that provide a detailed picture of the collision dynamics.^11,39,40^ A well-established approximation to this approach is called two-temperature theory (2TT).^41,42^ Here, the changes in the collision velocity are condensed into a set of temperatures, all of which are larger than the background temperature. First, the temperature describing the average collision velocity distribution is the effective temperature, *T*_eff_, and allows us to model the variation of the ion mobility coefficient, *K*, with the field strength:
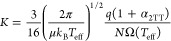
5a
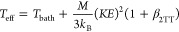
5b

Note that *T*_eff_ increases with increasing *E*. α_2TT_ and β_2TT_ are correction factors for higher-order approximations of 2TT, and *M* is the molecular mass of the collision gas. Second, a temperature *T*_L_ is defined describing the collision velocity distribution in the longitudinal (field) direction. *T*_L_ is used to calculate the longitudinal diffusion coefficient, *D*_L_, through the generalized Einstein relations (GER):^2^
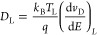
6Here, the derivative  is approximately equal to *K*. Importantly, *D*_L_ increases with increasing field strength through *T*_L_, resembling the fact that the additional translational energy imparted to the ion by the electric field increases the diffusion. See Section S1.2.2 of the Supporting Information for more details on the GER and the definition of *T*_L_.

In addition to the collision dynamics, higher reduced field strengths also affect the ion reaction dynamics. Namely, the increased collision energy increases the ion’s internal temperature, *T*_ion_, by means of inelastic collisions (ion heating). For example, it is commonly observed that the extent of fragmentation increases with increased reduced field strength, and the amount of ion-neutral clustering decreases.^43−49^ Ion heating is unique to IMS and is central to this work. Overall, this means that in order to correctly interpret the experimentally observed ATD, one has to consider the ion collision and reaction dynamics at the reduced field strength used in the instrument of interest. If the fields vary temporally, as in TWIMS, FAIMS, or TIMS, the situation further complicates.

In this work, we aim to systematically investigate how the ion collision dynamics and ion reaction dynamics influence the ATD (*t*_D_ and width/shape) as a function of the applied reduced field strengths. We utilize a DTIMS with variable but static reduced field strength (High Kinetic Energy IMS, HiKE-IMS^43^) first to allow for a deconvoluted investigation rather than turning to instruments with temporally varying fields. We investigate (1) nonreacting systems to study field-dependent collision dynamics, (2) “fast” chemistry, namely, ion–water clustering, to investigate shifts and broadening of the ATD, and (3) slowly reacting systems, namely, ion fragmentation, to study non-Gaussian peak shapes. We complement our experimental investigation with extensive modeling, combining a previously published model of ion mobility and ion chemistry^50,51^ with a new Monte Carlo treatment of the ion chemistry to be able to predict ATDs from first principles.

## Methods

### Experimental Methods

In order to measure the ATD and identify ion species, two different HiKE-IMS systems, i.e., a stand-alone HiKE-IMS^52^ and a coupling of a HiKE-IMS to a time-of-flight (ToF) MS as described in detail in previous publications^43,53^ are used with the most relevant operating parameters summarized in Table 1.

**Table 1:** Operating Parameters of the Two IMS Devices

parameter	HiKE-IMS	HiKE-IMS-MS
drift length	150.5 mm	150.5 mm
reduced electric field strength	30–120 Td	20–120 Td
pressure	14.7 mbar	14.3 mbar
temperature	80 °C	25 – 30 °C
injection time	3 μs	3 μs
approx. H_2_O content	70 ppm_V_	70 ppm_V_
ATD resolution	56 ns	3 μs

Briefly, in both systems, a corona discharge ion source is used to generate reactant ions that subsequently ionize the neutral analytes introduced into the reaction region. A tristate ion shutter^8^ is used to inject the ion population into a drift region, where the different ion species are separated based on their ion mobilities. In the stand-alone HiKE-IMS, a Faraday plate records the ion current at the end of the drift tube. HiKE-IMS-MS uses a second tristate ion shutter at the end of the drift tube to transfer selected ions into the ToF-MS, where they are separated based on their *m*/*z*. As typical HiKE-IMS peak widths (∼10 μs) and the duty cycle of the MS (∼50 μs) are on the same order of magnitude, direct MS sampling of the HiKE-IMS peaks is not possible. In recent work, we have demonstrated a revised shutter technique that allows for the acquisition of two-dimensional data of ion mobility vs *m*/*z* ratio.^52^ In this *2D-IMS-MS mode*, both the ion shutters in front of and after the drift region are opened for 3 μs with a fixed delay between the opening times to select the mobility of ions injected into the ToF-MS. Varying this time delay then enables recovering the full 2D-IMS-MS spectra. For details, see Section S5 in the SI.

All chemicals were purchased from Sigma-Aldrich, Germany, with a reported purity of ≥99%. Chemicals were used without further purification. Clean nitrogen was supplied using a nitrogen generator (NG5000A, Peak Scientific, U.K.) with an internal pressure swing absorber in series with an additional activated carbon filter (Supelcarb HC Hydrocarbon Trap, Supelco) and a moisture trap (Molecular Sieve 5A Moisture Trap, Supelco). The nitrogen provided contains <1 ppm_V_ of water and <0.5 ppm_V_ of oxygen. More information on the gas mixing system to provide the sample can be found in a previous publication.^51^ In all experiments, the relative humidity of the sample gas was adjusted to reach 10% (referenced to 293.15 K and 1013.25 hPa). The drift gas was not intentionally humidified. Note that the resulting water volume fraction inside the drift gas may well exceed 1 ppm_V_ due to diffusion through seals and tubing. Previous studies on HiKE-IMS-MS indicate that the residual water volume fraction should be around 70 ppm_V_.^54^

### Monte Carlo Integration

Previously, a first-principles model was presented, which is able to simulate the ion mobility and ion chemistry of a reacting system during the transit through an IMS device.^50,51^ For the ion chemistry, the relative populations of each species at a certain time, ***P***(*t*), are propagated in time via a state-transition matrix, i.e., through a Markov-chain process:
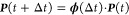
7Here, entry ϕ_*ij*_ describes the reaction probability from species *j* to species *i* in time interval Δ*t*, based on the respective rate constant (see below). For the ion motion, eq 4 is used, i.e., the ensemble moves a step of
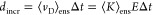
8a
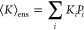
8bper Δ*t* through the device. Successive repetition of this process then yields temporal integration of the ensemble population distribution over the entire length of the drift tube, *L*.

To allow modeling of the actual ATD, even if the chemistry is “slow”, we switch to a particle-based (Monte Carlo, MC) approach. That is, we propagate individual particles through the drift tube and at each time step sample possible reactions. Conveniently, the same state-transition matrix, **ϕ**(Δ*t*), can be used since its columns represent the reaction probabilities of each species to react with any other species in the ensemble (see Section S1.1 of the SI for details). Each particle is propagated in space according to the mobility of the current identity instead of the ensemble mobility:
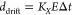
9

We also now include a random-walk treatment of diffusion along the drift tube length:^56^
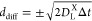
10That is, at each time step, the particle takes another step of magnitude  either in or against the drift direction (randomly sampled). Here *D*_L_^*X*^ is the longitudinal diffusion coefficient of the current particle identity, *X*. See Section S1.2.1 in the SI for justification of this treatment. Thus, in total, the particle is propagated a distance of
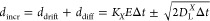
11per time step, Δ*t*. Note that both *K*_*X*_ and *D*_L_^*X*^ are evaluated at the applied reduced field strength, *E*/*N*. Once a particle reaches the total drift length, the propagation is terminated, and its final identity is recorded. Repeating this process for a large number of particles, *N*_p_, yields final ion populations and ion-specific ATDs. Thus, the MC method can describe the complicated coupling of the reaction and diffusion broadening of the ion cloud due to ion-specific mobility and diffusion coefficients. We note that for a nonreacting system, diffusion can be treated directly via Fick’s second law, i.e., taking the diffusion contribution to the peak width as . For a comparison between the Markov-chain method and the MC approach presented here, see Section S1.3 of the SI.

### Density functional theory (DFT), CCS, and Reaction Dynamics Calculations

To perform the modeling, ion structure, energetics, and mobility data are needed. To this end, we first optimize the geometry of all ion (cluster) structures involved using density functional theory (DFT), applying the ωB97X-D3(BJ)/def2-TZVPP^57−61^ level of theory. As outlined previously, we calculate the energy Hessian at the equilibrium geometry, project out internal rotations around ion–solvent binding axes (together with overall rotation and translation), and diagonalize the remaining sub-Hessian to obtain the vibrational frequencies.^51,55^ We further calculate atomic partial charges using the CHELPG scheme^62^ and also improve the electronic energy by performing single-point energy calculations applying the DLPNO–CCSD(T)/def2-TZVPP (TightPNO settings)^63,64^ level of theory. Electronic structure calculations are performed using ORCA (v5.0.4).^65,66^ Ion geometries and partial charges are then handed over to OpenBabel^67^ for automatic identification of atom classes within the MMFF94^68,69^ force field. This data is then passed over to MobCal-MPI 2.0^70^ to obtain mobility, CCS, *T*_eff_, and *T*_L_ data over the desired range of reduced field strengths (0–120 Td) using third-order 2TT. We highlight some limitations of this approach: First, even for molecules experiencing only a single conformer, the increase of *T*_ion_ with increasing reduced field strength leads to stronger vibrations and thus to an enlargement of the CCS. This effect is small for rigid molecules but can be large for clusters.^25^ Second, if multiple conformers exist, then the populations of these conformers can change with *T*_ion_. For example, flexible molecules will enlarge/unfold upon heating.^71^ We deliberately chose rigid test systems to avoid this issue here, but for more flexible molecules, this effect needs to be considered. Third, as MobCal-MPI 2.0 calculates elastic CCS, all inelastic effects are ignored, which likely has a significant effect on flexible molecules at higher reduced field strength.^39,72,73^ Lastly, as we have no explicit treatment of inelasticity, we approximate *T*_ion_ ≈ *T*_eff_.

The systems studied here experience both loose (e.g., cluster dissociation reactions) and tight (e.g., rearrangement reaction) transition states (TSs). To construct the state-transition matrix, field-dependent rate constants for all possible reactions are modeled according to the respective TSs. Loose TSs are treated according to SACM theory^74^ as described previously,^50^ assuming either an ion-dipole or an ion-induced dipole potential (see Section S1.1 of the SI for details). Tight TSs can be directly modeled using the Eyring equation^75^ using the TS structure and energetics. These are obtained by first mapping out the reaction path using the nudged-elastic band (NEB) method^76^ as implemented in ORCA, followed by a TS optimization starting from the highest energy point of the found path.

In total, we obtain ion energetics and thermochemistry, mobility data (collision dynamics), and reaction kinetic data (reaction dynamics) from first principles. All structures are available in the ioChem-BD database (see Data Availability Statement).

## Results and Discussion

### Field-Dependent Mobility and Diffusion of Nonreacting Systems

First, we investigated the influence of the field dependency of the mobility and diffusion (collision dynamics) on the ATD without ion transformation processes. To this end, we turned to protonated 2,6-Di-*tert*-butylpyridine (2,6-D*t*BP), a prime ion mobility standard showing negligible clustering with water.^77^ Thus, we measured and computed the ATD of 2,6-D*t*BP between 20 and 120 Td and obtained the drift time, *t*_D_, and the peak width (full width at half-maximum), *w*_0.5_, through fitting a Gaussian to the ATD. See Figure S4 for exemplary ATDs. In both experiment and simulation, the reduced mobilities, *K*_0_, can then be obtained from the fitted Gaussian via
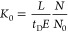
12where *N*_0_ is the Loschmidt constant. For comparison to the MC-determined diffusion width, we also compute the diffusion width analytically, which is readily done for a nonreacting system:
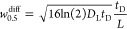
13

For better comparison to experimental data, both the analytically calculated and MC-determined diffusion widths are subsequently convoluted with an initial ion package width (assuming a Gaussian) according to
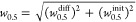
14whereby we set the standard deviation of the initial Gaussian equal to the standard deviation of a rectangular ion package created by the shutter opening time (). Effects like space charge expansion and broadening through the transimpedance amplifier^7,8^ are ignored for simplicity.

**Figure 1:**
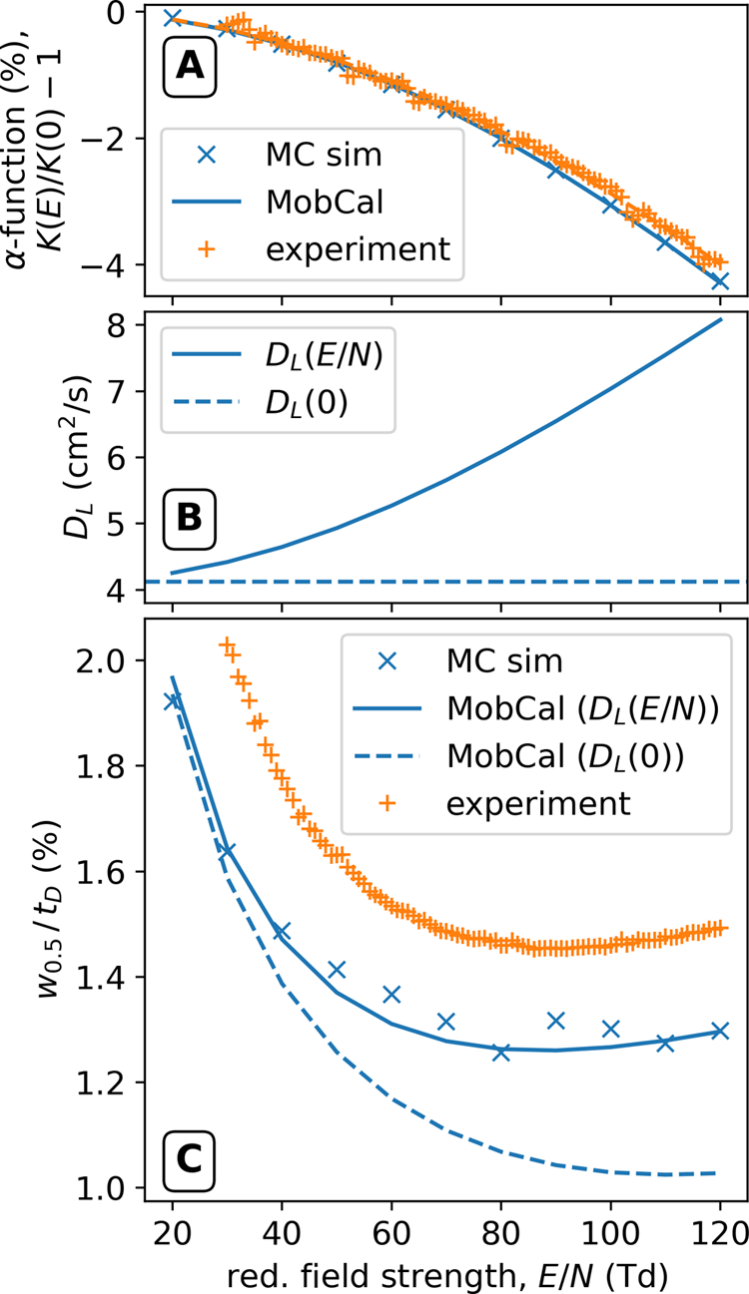
(A) α-Function, (B) longitudinal diffusion coefficient, *D*_L_, and (C) relative peak width, *w*_0.5_/*t*_D_, of protonated 2,6-Di-*tert*-butylpyridine obtained from measurement (orange) and calculation (blue; solid and dashed lines from MobCal-MPI 2.0, points from MC simulations using *N*_p_ = 4000) between 20 and 120 Td.

Figure 1 shows the change in mobility (α function), diffusion coefficient, and relative peak width, *w*_0.5_/*t*_D_, between 20 and 120 Td obtained experimentally using the stand-alone HiKE-IMS (used for more accurate sampling of the ATD) and via simulations. As can be seen in Figure 1A, the α-function of 2,6-D*t*BP decreases by a few percent as the reduced field strength increases, which is the expected behavior for an ion of this mass and low charge state.^78^ The computed α-function is in excellent agreement with the experiment. While the reduced low-field mobility is predicted to be too high (sim.: 1.519 cm^2^/(V s), expt.: 1.443 cm^2^/(V s), or in terms of CCS, sim.: 131.2 Å^2^, expt.: 138.1 Å^2^), this deviation is still within the errors reported for MobCal-MPI 2.0.^70^ Note that the α-function obtained via eq 12 from the MC data is virtually identical to the ones obtained through MobCal-MPI 2.0 (solid line). As the MobCal-MPI 2.0 data are used as input for the MC simulations (eq 9), this shows the internal consistency of the MC framework.

The field dependency of the reduced mobility can introduce uncertainties when calibrating instruments such as TIMS or TWIMS with mobilities determined under low-field conditions: Operating at elevated reduced field strengths, the calibrants will experience different mobility (according to their α-function) as compared with their low-field mobility. The same is true for the analytes in question, and only if the α-functions of the analyte and calibrant were the same this effect would cancel out. For this reason, it is usually recommended that calibrants of the same chemical class and charge state as the analytes should be used to minimize the difference in the α-function between calibrants and analytes.^1^ Because the change of mobility with field strength strongly depends on the structure and charge distribution of the ion,^25,78^ it is difficult to make a general statement about the direction or magnitude of this nonlinearity. Consequently, measuring^79^ or computing the α-function of a target analyte can help in choosing suitable calibrants (with known α functions), decreasing uncertainties in calibration procedures.

As can be seen in Figure 1B, the ions diffusion coefficient, *D*_L_, increases significantly with increased reduced field strength. Importantly, while the mobility of 2,6-D*t*BP changes only by a few percent, the diffusion coefficient increases by more than a factor of 2 between 20 and 120 Td. This highlights the fact that the additional energy imparted on the ions through higher reduced field strengths, expressed through the temperature *T*_L_ (cf. eq 6), leads to increased ion diffusion.

To investigate the effect of increased ion diffusion on the peak width, we studied the relative peak width, *w*_0.5_/*t*_D_. The relative peak width is equivalent to the inverse resolving power and thus contains the same information. The experimental data in Figure 1C can thus be viewed as analyzing the resolving power of the instrument. However, as our theoretical model only contains contributions from diffusion and initial peak width, we do not claim to predict actual resolving powers, and by plotting relative peak widths instead, this distinction is hopefully clear. In a future publication, we aim to provide a model predicting the actual resolving power in DTIMS, including effects from field-dependent diffusion and the initial width of the ion cloud and transimpedance amplifier.

Higher reduced field strengths decrease the drift time of the ions according to *t*_D_ ∝ (*E*/*N*)^−1^. As such, the ions have less time to diffuse in the longitudinal (field) direction, and eq 13 predicts *w*_0.5_^diff^/*t*_D_ ∝ (*E*/*N*)^−1/2^ when considering a constant diffusion coefficient (low-field limit). That is, the relative peak width decreases with increasing reduced field strength. On the other hand, the temporal initial pulse width, *w*_0.5_^init^, remains constant, leading to a dependency of *w*_0.5_^init^/*t*_D_ ∝ (*E*/*N*)^+1^. Overall, this leads to an initial decrease of the peak width (since diffusion usually contributes more strongly at lower reduced field strengths), which eventually leads to an increase through the contributions from *w*_0.5_^init^. This can be seen in Figure 1C when viewing the dashed line. More details can be found in ref (80). However, the increase of the diffusion coefficient with increasing reduced field strength (cf., Figure 1B) changes the diffusion contribution to the relative peak width. Specifically, in the limit of very high reduced field strengths, a dependency of *w*_0.5_^diff^/*t*_D_ ∝ (*E*/*N*)^+1/2^, i.e., an *increase* of the relative peak widths with the reduced field strength can be found, despite shorter drift times (see Section S1.2.3 of the SI for the derivation and further details). As a result, the overall relative peak width (taking together diffusion and initial peak width contributions) has a minimum at a much smaller *E*/*N* and is not as low as compared to a constant diffusion coefficient. This can be seen by comparing the solid and dashed lines in Figure 1C. In other words, above a certain *E*/*N*, ion diffusion negatively impacts relative peak widths (and thus resolving powers) despite shorter drift times. We note again that the MC data for the relative peak widths match the analytical expression well, validating the used random-walk treatment of diffusion (eq 10), in particular, that the number of time steps used is sufficient to accurately model the spread of ions for the given drift times.

Comparing the computed data to the experimental relative peak width, we find that the treatment using a field-dependent diffusion coefficient (solid line and MC data) yields better agreement than when using a constant diffusion coefficient. Deviations between the experiment and computations are likely due to the fact that the experimental peak widths also include contributions from space charge expansion of the ion cloud and the transimpedance amplifier of the instrument, additionally increasing the peak width over the modeled one. Still, based on both the quantitative and qualitative (shape of the curves) agreement, these data support the notion that the increase of ion diffusion with increased reduced field strength significantly affects peak widths and should be considered when operating above the low-field limit.

It should be noted that we confirmed that 2,6-D*t*BP did not form any ion–water clusters both experimentally and computationally. See Section S2 of the SI for details.

### Dynamic Equilibria—Ion–Water Clusters

After showing how the ATD of a chemically inactive species evolves as a function of reduced field strength, we turn to a chemically active system showing quick equilibration, namely, protonated methanol clustering with water. As stated above, we estimate the background water concentration in the drift region to be around 70 ppm_V_, giving H^+^(MeOH) the opportunity to form water clusters. In fact, we studied this system previously^51^ and observed both the monohydrate, H^+^(MeOH)(H_2_O), and the dihydrate, H^+^(MeOH)(H_2_O)_2_. Further, since neutral methanol is diffusing into the drift region from the reaction region (see Figure S6 for concentration profile), the proton-bound methanol dimer H^+^(MeOH)_2_ and its hydrate H^+^(MeOH)_2_(H_2_O) are observed in low abundance as well.

**Figure 2:**
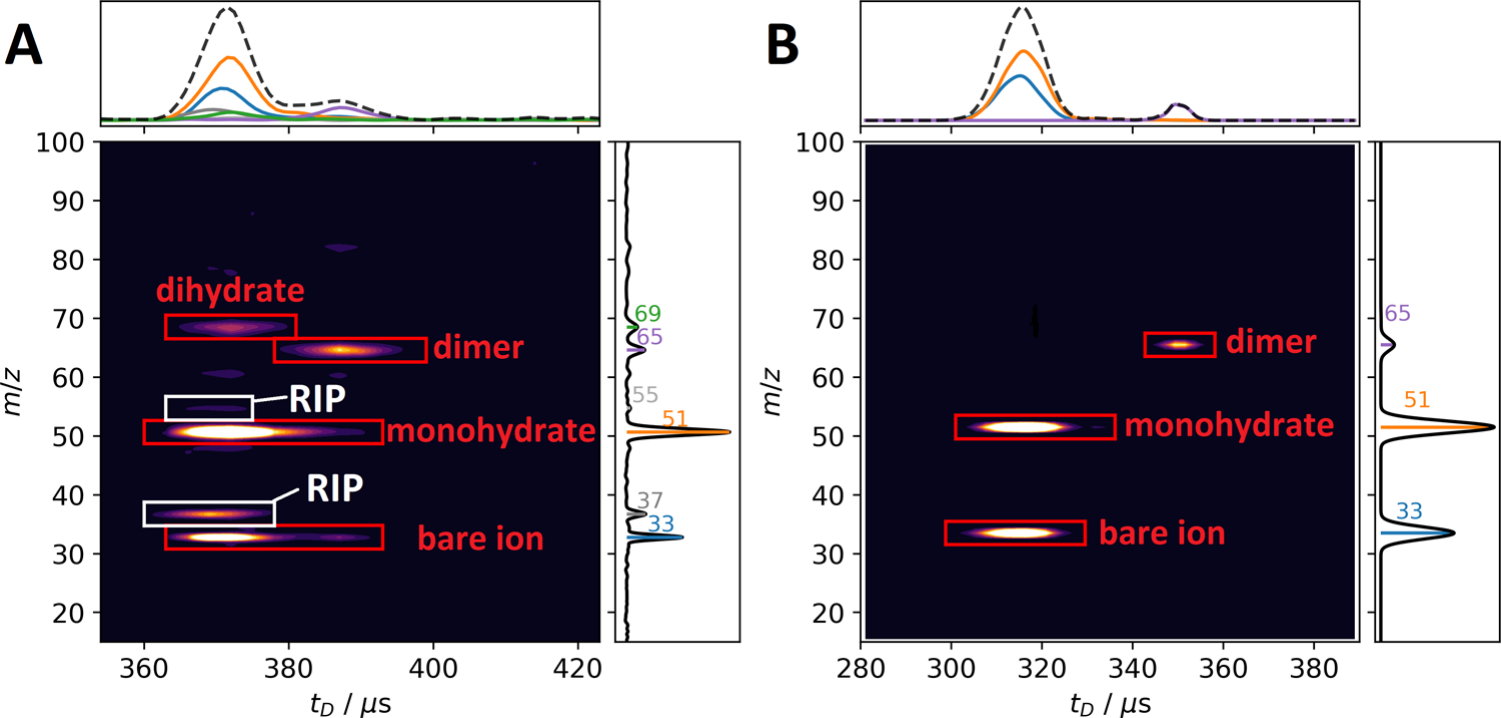
2D-IMS-MS data for the [MeOH_2_^+^ + *n*(H_2_O) + *m*(MeOH)] system, (A) as measured with HiKE-IMS-MS and (B) predicted by the MC method presented here. The reduced field strength in the drift region was 70 Td.

Figure 2 shows the 2D-IMS-MS data for this system at a reduced field strength of 70 Td as obtained from the experiment and MC data. Note that the experimental data (Figure 2A) also shows the reactant ion peak (RIP), here consisting of the H^+^(H_2_O)_2_ species (*m*/*z* 37), which is not considered in the calculations. Consistent with our previous publication,^51^ at 70 Td, the monohydrate of protonated MeOH is the most abundant species, followed by the bare ion. The dihydrate and proton-bound dimer are visible in low abundance. This is well reproduced by the MC simulations (Figure 2B), although the low abundance of dihydrate is missing and the absolute drift times are smaller in the modeling. The latter is likely due to the fact that MobCal-MPI 2.0 was not parametrized for small ions like this, yielding a somewhat larger error in the calculated mobilities.

Most importantly, the experimental and simulated data show that all hydrates of protonated methanol arrive at virtually the same drift time, and only through the additional MS information, i.e., mass-resolved ATDs, can we deconvolute the total ATD into ATDs of different species. This is direct evidence that they interconvert many times during the transit through the drift tube. Using the simulations, we can determine the arrival times for each of the hydrates (*n* = 0,1,2) as if no reactions would take place. At 70 Td, these are calculated as 296, 326, and 356 μs, respectively, compared to the 315 μs observed in the MC treatment *with* dynamic clustering/declustering (see Figure S7). In particular, compared with the expected arrival time of the bare ion, the observed (i.e., MC simulated) arrival time is shifted to longer drift times. This can be pictured as the ion having a (on average) larger CCS through the clustering with water. This is analogous to an additional retention factor in chromatography.^14^ The fact that the proton-bound dimer (and its hydrate) arrives at a later drift time hints at much slower ion chemistry for the interconversion between monomer and dimer. Indeed, Figure S8 shows the number of reaction events occurring during the drift, as determined by the MC simulations, and while there are around 35 water-related reaction events occurring per ion, less than 10 reaction events occur related to neutral methanol. This is not sufficient for the merging of peaks, and only a small elevation of the baseline between the monomer (hydrates) and the dimer peak is observable.

We want to highlight that ion-cluster equilibria depend on the reduced field strength^19^ and water/solvent vapor content.^20^ If these parameters vary from day to day, this can have negative effects on the reproducibility of derived mobility coefficients and CCS. On the other hand, shifts in the ATD through dynamic clustering have also been used in the past to increase separation between compounds, as the amount of clustering depends on the ion-specific binding strengths.^22^ When instruments with oscillating fields are used (FAIMS, TWIMS for separation and/or focusing, TIMS for focusing), the ensemble mobility will constantly vary depending on the cluster population distribution at the sampled reduced field strengths.^50,78^

**Figure 3:**
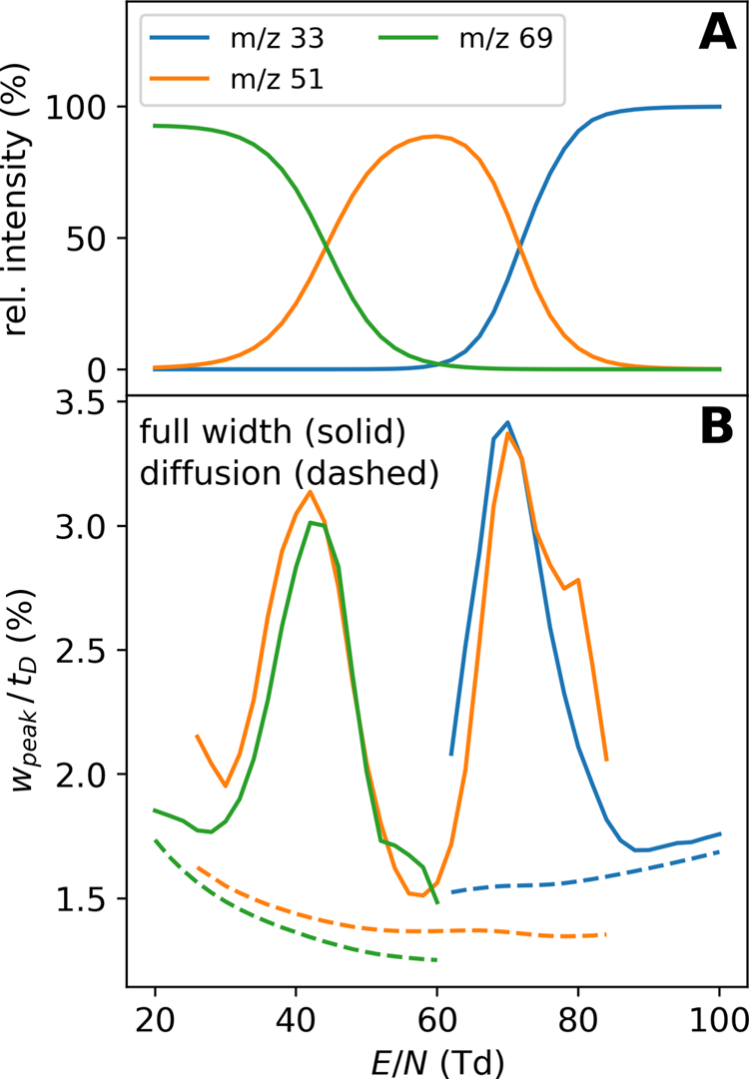
Peak broadening through ion-cluster transitions: (A) population of different hydrates of protonated methanol and (B) relative peak width of the mass-selected ATDs. All data were obtained through the MC modeling (*N*_p_ = 4000).

In addition to the *position* of the ATD, ion-cluster chemistry can also alter the *width* of the ATD. Importantly, as the reduced field strengths influence the ion-cluster equilibria, it should also affect the peak widths. Using our MC model, we conducted simulations of this ensemble over a range of 20–100 Td and computed the relative peak widths, *w*_0.5_/*t*_D_, again by fitting a Gaussian to the (unimodal) ATD. For simplicity, the initial ion distribution is assumed to have zero width. Together with the relative populations of each cluster species, these data are shown in Figure 3. We also show what relative peak width one would expect for the individual cluster species without any clustering reactions, i.e., purely through diffusion broadening (dashed lines in Figure 3B). This can be considered a baseline/minimum relative peak width. Indeed, we can observe that the MC relative peak widths are in good agreement with this baseline whenever the ion ensemble is dominated by only one species (dihydrate at 20 Td, monohydrate at 60 Td, and bare ion above 90 Td). However, when there is a cluster transition occurring, the relative peak widths increase significantly compared with the diffusion baseline. This means that when the instrument is operated at a reduced field strength, where ions experience many back-and-forth reactions, peak broadening will be observed, resulting in a decrease in resolving power. An example of ATD (simulated) showcasing this broadening effect can be found in Figure S7 of the SI.

It is interesting to note that while the mobility of a dynamic ensemble is a weighted average of the individual ion mobilities, the peak width is not a weighted average of the “diffusion baseline widths” but instead significantly larger. This highlights that the ion chemistry adds additional inhomogeneity to the individual ion trajectories. That is, on top of the different paths taken by individual ions caused by random collisions (diffusion), random reaction events, altering the mobility, further broaden the ion cloud. This is true not only for dynamic clustering with solvents but also when dealing with ions that can readily interconvert between two or more conformers. In turn, comparing the width of the ATD to what would be expected from diffusion alone (considering its field dependency) could reveal information about the structural inhomogeneity of the studied ion. As stated in ref (18), the width of the ATD is usually “under-exploited”.

### Non-Gaussian ATDs—Slow Ion Chemistry

**Scheme 1:**
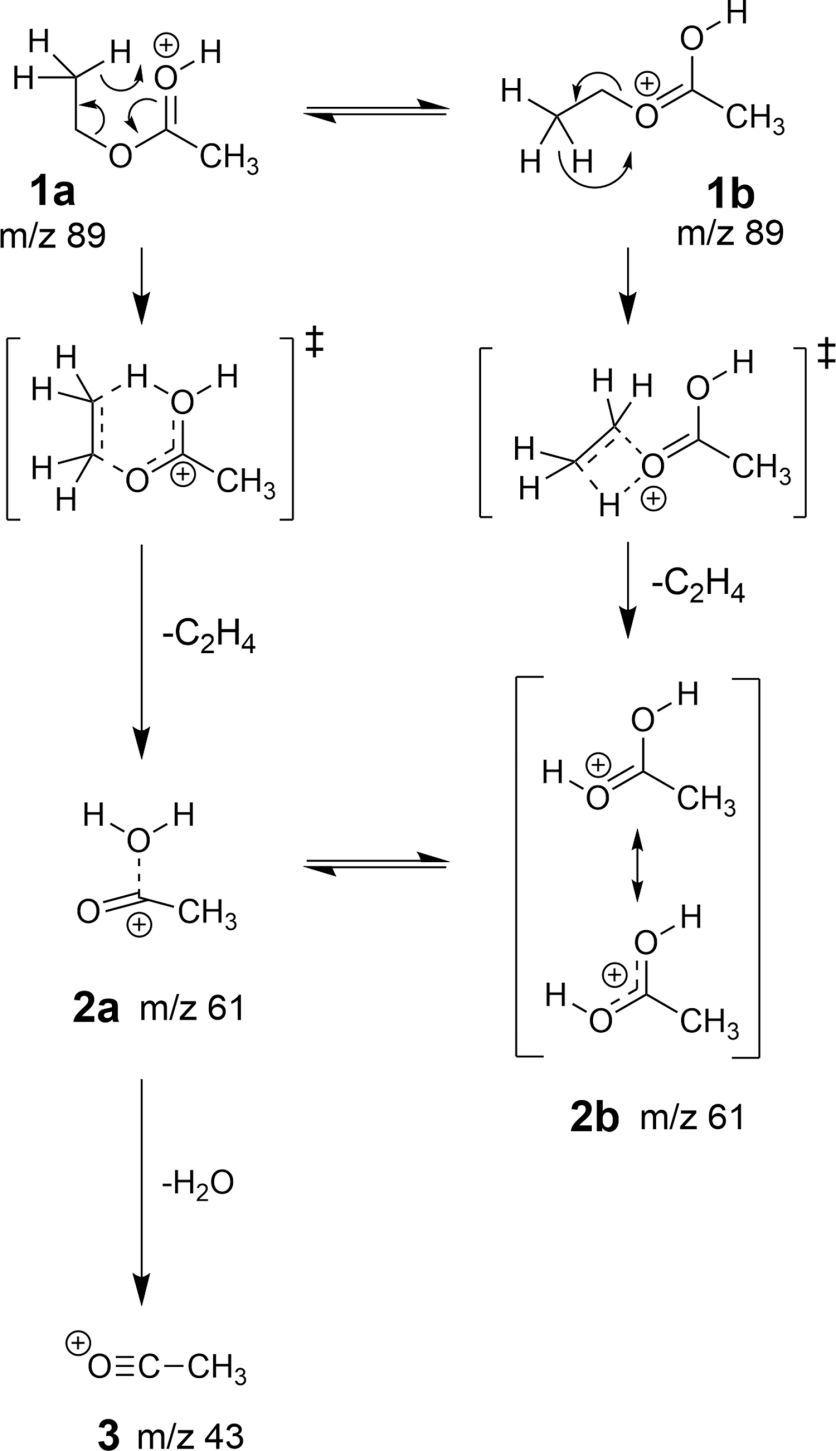
Different Fragmentation Pathways of Protonated Ethyl Acetate McLafferty rearrangement as proposed in ref (81) (pathway **1a** → **2a**) and rearrangement found in this study and in ref (82) (pathway **1b** → **2b**).

If the chemical transformation rates are of the same order of magnitude as the drift time, non-Gaussian ATDs will be observed, independent of whether the chemical transformation is clustering/declustering, fragmentation, or conformational dynamics.^15,18^ For a detailed investigation of the effect of elevated reduced field strengths on fragmenting systems, we turn to ethyl acetate (EtOAc), observed as a protonated form, which is known from PTR-MS to undergo fragmentation at high reduced field strengths.^81^ In particular, as shown in Scheme 1, the protonated ion (**1**, *m*/*z* 89) first undergoes loss of ethene (C_2_H_4_), yielding H_3_C–CO_2_H_2_^+^ (**2**, *m*/*z* 61), which further loses water, yielding H_3_C–C≡O^+^ (**3**, *m*/*z* 43). Previously, it was thought that the first fragmentation step proceeds through a McLafferty rearrangement (**1a** → **2a**).^81^ However, we found in our DFT modeling that the proposed species **2a**, being doubly protonated at one oxygen, is only weakly bound (C–OH_2_ distance of 2.39 Å, almost linear O=C–CH_3_ angle of 171°). It seems unlikely that this loosely bound complex is the dominant species in PTR-MS. Instead, a different rearrangement (**1b** → **2b**) can cleave the ethene moiety, leaving one proton at each oxygen atom (**2b**). This yields a more stable fragment and shows a comparable barrier as the earlier proposed McLafferty rearrangement (Δ*G*_a_^⌀,‡^ = 136.5 kJ/mol vs Δ*G*_b_^⌀,‡^ = 133.5 kJ/mol). Interestingly, the same rearrangement was recently found for an alternative class of thermometer ions studied in FAIMS.^82^**2b** can subsequently interconvert to **2a** (Δ*G*^⌀,‡^ = 185.8 kJ/mol) for further fragmentation to **3**. While these barriers might seem high, the ion temperatures at high reduced field strengths largely exceed the background gas temperature, rendering these mechanisms feasible. More details on the energetics and structures of the fragmentation mechanism can be found in Section S4.1 of the SI.

**Figure 4:**
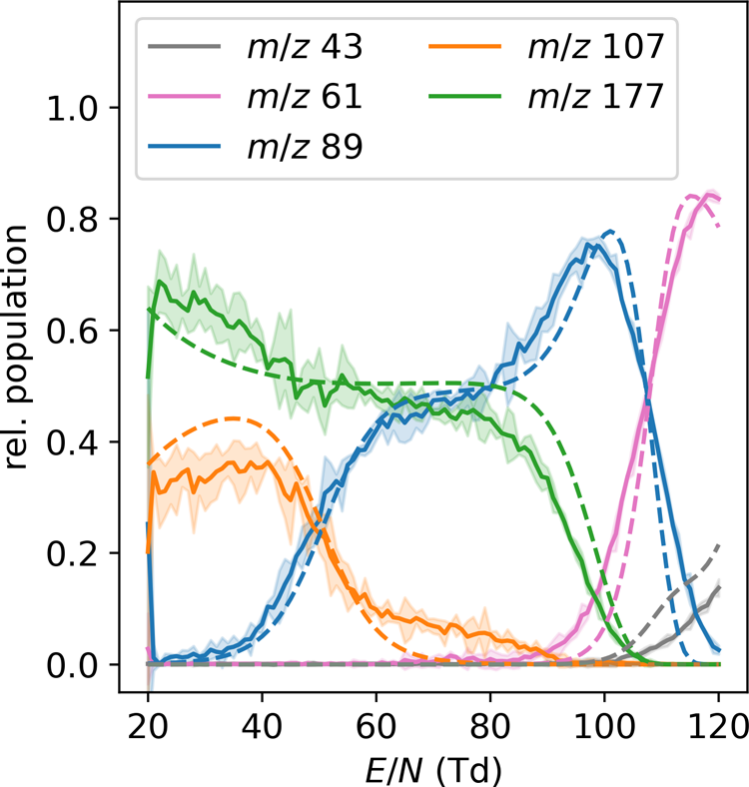
Relative population of all species of the EtOAc system as a function of reduced field strength as measured by HiKE-IMS-MS (solid lines with error band (2σ, *n* = 3)) and calculated through the MC framework (dashed lines). Alongside the bare ion (*m*/*z* 89) and its fragments (*m*/*z* 61, 43), also the monohydrate (*m*/*z* 107) and proton-bound dimer (*m*/*z* 177) are visible.

To gain an overview of the fragmentation behavior of protonated EtOAc in HiKE-IMS (as opposed to PTR-MS), we first recorded the abundance of each species involved as a function of reduced field strength. This also allows us to validate whether the MC modeling predicts the correct ion chemistry as a function of reduced field strength.^51^ As can be seen in Figure 4, the fragments known from PTR-MS appear above ca. 100 Td in successive order, as expected from the reaction mechanism (Scheme 1). The high reduced field strengths thus drive the ion fragmentation. Importantly, **3** (*m*/*z* 43) appears at reduced field strengths even higher than that of **2** (*m*/*z* 61), clearly showing that **2** is a stable intermediate. This matches our modeling, where the large barrier between **2a** and **2b** prevents immediate dissociation of **2b** to **3**. Importantly, measuring fragmentation efficiency as a function of reduced field strengths thus yields detailed information on ion stabilities. Below 100 Td, we also observed significant amounts of H^+^(EtOAc)(H_2_O) (*m*/*z* = 107) and H^+^(EtOAc)_2_ (*m*/*z* = 177). The data closely resembles what we have observed previously for protonated acetone,^51^ and can be explained by a ligand-switching mechanism between the monohydrate and proton-bound dimer and their different stabilities (see Figure S12).

After some fine-tuning of the fragmentation reaction rates (see Section S4.3 of the SI for details), the modeled relative populations closely match the observations over the whole range of reduced field strengths very well. This gives us confidence that the most important aspects of the ion chemistry involved are well captured in the model and that it can be applied to study ATDs next.

**Figure 5:**
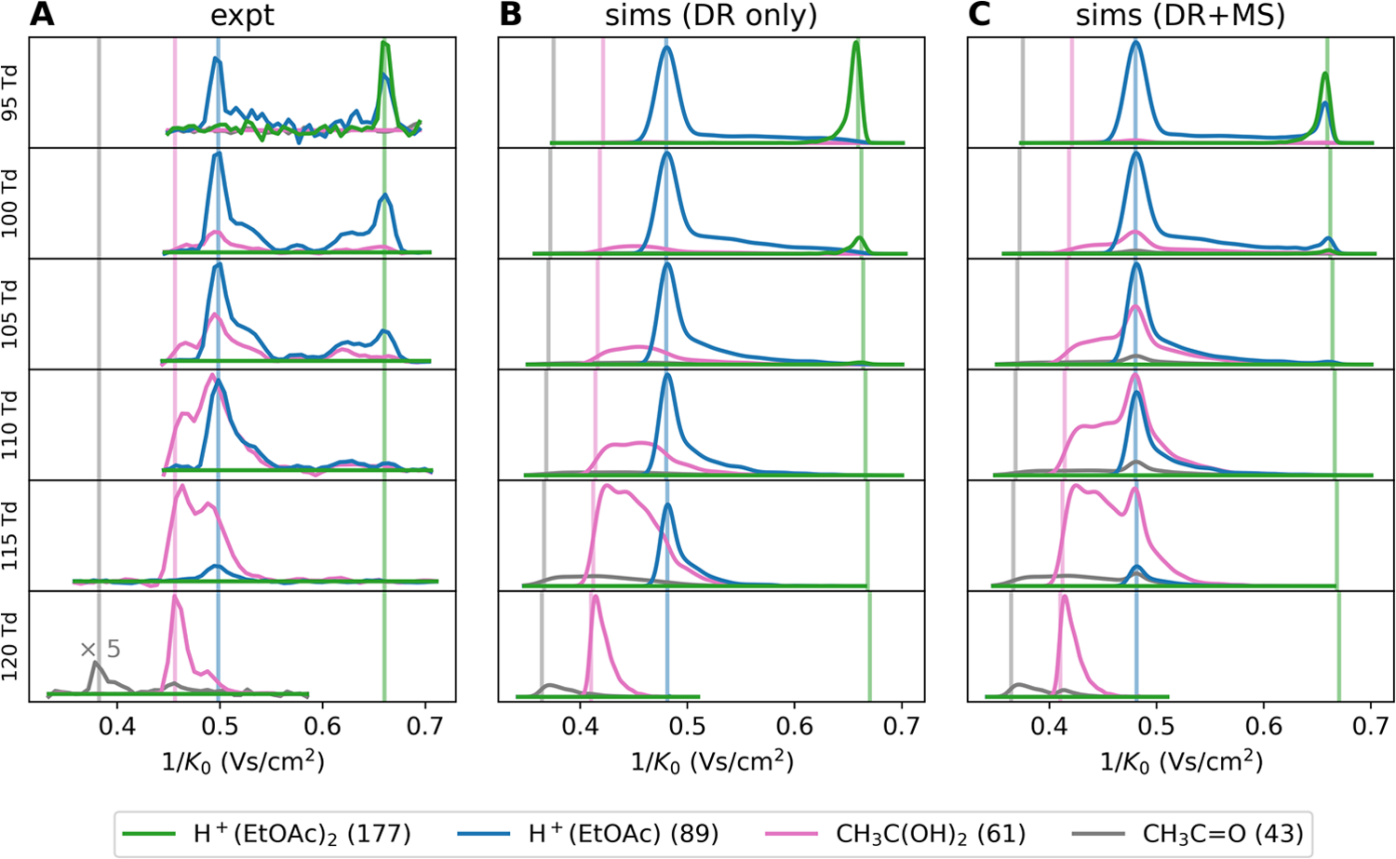
Mass-resolved ATDs of the EtOAc system between 95 and 120 Td. The *m*/*z* of the species are given in parentheses in the figure legend. (A) experimental spectra, (B) simulated ATDs for the drift region (DR only), and (C) simulated ATDs for the drift region + MS transfer region (DR + MS), whereby the latter includes further reaction time in the MS transfer region. Vertical lines indicate the reduced mobilities of the individual species.

As we are mostly interested in the fragmentation of H^+^(EtOAc), we recorded 2D-IMS-MS spectra between 100 and 120 Td using HiKE-IMS-MS. These can be found in Figure S16. We subsequently extracted mass-resolved ATDs for all signals observed in the MS, i.e., the proton-bound dimer (*m*/*z* 177), the bare ion (*m*/*z* 89), and its fragments (*m*/*z* 61 and 43). These ATDs are shown in Figure 5A and reveal multiple features, each showing complex non-Gaussian peak shapes, which vary with the reduced field strength. In particular, multiple unique mobilities can be identified, which is in contrast to the ensemble mobility observed in the MeOH system. These unique 1/*K*_0_ values, associated with the respective species, are shown as vertical lines in Figure 5A. At different reduced field strengths, different species dominate the overall spectrum. For example, the amount of parent ion observed decreases with *E*/*N*, whereas the amount of *m*/*z* 61 fragment increases. This is consistent with that in Figure 4.

At 95 Td, two main features are observed in the mobility spectrum: a high mobility feature consisting of the H^+^(EtOAc) species and a lower mobility feature that shows contributions from both the monomer and proton-bound dimer of EtOAc. In fact, as the reduced field strength increases, the proton-bound dimer quickly vanishes, and the lower mobility feature solely consists of the bare H^+^(EtOAc) ion. This will be discussed further below. Interestingly, the monomer feature shows strong tailing toward the dimer feature, which is commonly observed in stand-alone IMS when proton-bound dimers dissociate slowly over the course of the drift tube.^34^ Notably, the lower mobility feature also shows fronting toward the higher mobility feature. This is again known behavior from stand-alone IMS when proton-bound dimers are formed during the transit through the drift region.^31^ It is, however, unusual to see both fronting and tailing as one rate constant should always be larger than the other, and thus, either net dissociation or net formation of the proton-bound dimer should be observed. We were able to reproduce this behavior in the simulations (see Figure S14) by assuming a nonhomogeneous concentration of neutral EtOAc in the drift tube, similar to the MeOH system. As this is not the main focus of this article, the interested reader is referred to Section S4.2 of the SI for further details.

More interestingly, the higher mobility feature shows a transition from the parent ion toward the *m*/*z* 61 fragment as the reduced field strength is further increased. Specifically, the intensity of the parent ion at its reduced mobility decreases with field strength, while the intensity of the *m*/*z* 61 fragment increases when viewed at its reduced mobility. Notably, we also observe a significant amount of fragments at the reduced mobility of the parent, first increasing in intensity and then decreasing. This is comparable to the parent ion being observed at the mobility of the proton-bound dimer. Overall, this results in a very broad feature that, at specific reduced field strengths, even shows a bimodal distribution with strong tailing toward lower mobility values.

To understand this complex peak shape and its evolution, we turn to MC simulations. Simulated mass-resolved ATDs are shown in Figure 5B,C. Focusing on the simulated data labeled “sims (DR only)” first, we can see that the model nicely reproduces the reduced mobility of the different species (albeit with some minor deviations), the evolution of the intensities with increasing reduced field strength, and the overall broadness and width of the ATD. Importantly, strong tailing of the *m*/*z* 61 fragment peak toward the parent ion peak is observed. At high reduced field strengths, we further observe the *m*/*z* 43 fragment in low abundance, which is consistent with the measurements. The simulations reproduce what is known from theory,^15,34^ namely, that the tailing is caused by slow fragmentation over the course of the drift tube with a fixed rate, leading to an exponential decay of the parent, see Figure S11. It is important to note that the fragmentation is not fully completed when the ion cloud reaches the end of the drift tube, as still significant amounts of parent ions are observed (except at 120 Td). Thus, a longer drift tube would yield different mass-resolved ATDs. Since the fragmentation rate increases with increasing reduced field strength, more fragmentation is observed at higher *E*/*N*, even though the total drift time is shorter. Thus, the intricate balance between the fragmentation rate and the time that the ions have to react (i.e., the drift time) gives rise to the complex ATDs.

Interestingly, however, the simulations “sims (only DR)” do not reproduce the *m*/*z* 61 fragment peak observed at the reduced mobility of the parent or the monomer peak at the reduced mobility of the proton-bound dimer. In order to explain this behavior, it is important to note that additional fragmentation can occur in the transfer region of the MS. This has been observed before, e.g., on commercial TWIMS instruments,^35^ and is usually attributed to the harsh conditions in the differential pumping stages of the transfer region. In HiKE-IMS-MS, the transfer is designed to be very soft. Hence, in a first approximation, we model the ion chemistry in the transfer region by assuming that the ion temperatures remain the same as those in the drift region (no additional heating or cooling). Thus, the ions merely have a longer time to fragment, namely, 50 μs, which is roughly the time delay between the second ion gate and the ToF-MS pusher trigger. For situations in which the ion chemistry is already equilibrated (e.g., fast clustering or completed fragmentation), this would not change the relative populations. Here, however, the fragmentation processes are slow enough that they have not reached equilibrium at the end of the drift tube (kinetic shift^83^). Thus, giving the ions more reaction time significantly alters the populations, and the resulting ATDs (shown in Figure 5C, labeled “sims (DR + MS)”) then match the observed distributions very well. In particular, a portion of the ions that do not fragment in the drift tube then fragments in the transfer region, yielding lower mass peaks at reduced mobilities of the respective precursor species.

Overall, ion transformation processes, such as fragmentation, can occur not only during the IMS separation but also in the transfer to the MS in the case of an IMS-MS coupling. Importantly, elevated reduced field strengths can drive fragmentation processes and yield complex peak shapes even if mass-resolved ATDs are considered. To correctly interpret these spectra and harness important information about ion chemistry and ion stability, simulations such as the ones presented here can be very helpful.

It should be noted that the ATDs measured by TIMS and TWIMS or the ionograms measured by FAIMS for fragmenting systems will vary from the ones shown here for DTIMS as the exact details of the ion mobility separation, i.e., the used fields, their strengths, and temporal evolution, the influence of diffusion, etc., differ. These will be studied in future work.

## Conclusions

In this work, we have studied how elevated reduced field strengths alter the collision dynamics and reaction dynamics of ions in ion mobility spectrometry (IMS) and thus influence the position, width, and shape of their arrival time distributions (ATDs). Our investigation was conducted using a home-built drift tube IMS (coupled to a home-built mass spectrometer) and extensive first-principles Monte Carlo modeling. This allowed us to obtain absolute (without the need for calibrants) and mass-resolved peak positions and widths under very controlled conditions. The modeling, in turn, was used to obtain in-depth insights into the underlying processes, helpful for interpreting the data. The field dependency of the ion dynamics is particularly important, as modern IMS devices, such as TWIMS and TIMS, often operate at elevated reduced field strengths.

In terms of collision dynamics, we reviewed how elevated reduced field strengths alter both ion mobility and ion diffusion. While the ion mobility varies by only a few percent but in an ion-specific manner (giving rise to the separation capability of FAIMS), ion diffusion significantly increases with increasing reduced field strength. This has implications for both where the peak is expected and how broad it is. Namely, if an analyte has a very different α-function from the used calibrants (in instruments that require calibration, e.g., TWIMS), the derived CCS might show a significant error. Further, the broadening of the peaks through increased diffusion will lower the resolving power. First-principles modeling of the field dependency of ion mobility and ion diffusion is readily performed and can help estimate the magnitude of these effects for the analyte and instrument at hand.

On top of (and heavily influenced by) the collision dynamics, we showed that the ion reaction dynamics, as driven by the reduced field strengths, can also have a significant effect on the observed ATDs and what species are detected by MS. Generally, one should differentiate between “fast” and “slow” ion chemistry depending on whether the chemical processes are much faster or comparable to the ion mobility separation time scales but should keep in mind that rate constants vary with the applied reduced field strengths. We showed that fast ion chemistry (e.g., reversible conformational flexibility or clustering with surrounding solvent vapor) shifts and potentially broadens the ATD. This can advantageously be used to increase separation,^22^ but can also lower reproducibility when the influencing parameters (temperature, concentration, reduced field strength) vary from experiment to experiment. Slow ion chemistry (e.g., fragmentation and unfolding) will usually yield non-Gaussian ATDs, which complicate the spectrum. Again, this can be used as an advantage when studying ion chemistry or to increase selectivity (e.g., when specific fragments are studied) and can also lead to poor resolving power, difficult peak fitting, or low ion intensity (when fragmentation is overlooked). Modeling the ion reaction dynamics (on top of the collision dynamics) can help to harness important information on the analyte’s ion chemistry and avoid false interpretations of spectra. As this modeling involves reaction dynamics on top of collision dynamics simulations, significantly more effort is required.

In future work, we will apply the presented considerations and modeling to other IMS techniques, such as TIMS, TWIMS, and FAIMS.

## Data Availability

Structures of all calculated ions (including transition states) and neutrals are available through the ioChem-BD database.^84,85^ Specifically, the MeOH system can be found at 10.19061/iochem-bd-6-200 (from ref (51)), while structures corresponding to 2,6-D*t*BP and EtOAc can be found at https://doi.org/10.19061/iochem-bd-6-352.

## Supporting Information Available

Details on the Monte Carlo framework, each system studied, and the experimental setup (PDF)
