# Supplementary material for: On the Arrival Time Distribution of Reacting Systems in Ion Mobility Spectrometry
Source: Anal Chem. 2024 Jul 15;96(30):12433–43. doi: 10.1021/acs.analchem.4c02010 (PMC11295131; doi:10.1021/acs.analchem.4c02010)
Supplement: Supplementary file 1 — ac4c02010_si_001.pdf [file ac4c02010_si_001.pdf]

Supporting Information

On the Arrival Time Distribution of Reacting Systems in Ion Mobility Spectrometry

Alexander Haack[a]*, Christoph Schaefer[a], Stefan Zimmermann[a]

[a] Department of Sensors and Measurement Technology, Institute of Electrical Engineering and Measurement Technology, Leibniz University Hannover, 30167 Hannover, Germany

* Correspondence to: haack@geml.uni-hannover.de

Table of Contents

S1: Details about the Monte-Carlo propagation 2

S1.1: Reaction Sampling via the State-Transition Matrix 2

S1.2: Diffusion of Particles along the Drift Length 5

S1.2.1: Diffusion according to Fick’s second law 5

S1.2.2: Diffusion coefficients at arbitrary field strengths 6

S1.2.3: Diffusion broadening for varying fields 6

S1.3: Comparison to Markov-chain Method 8

S2: Additional Data for 2,6-D*t*BP 11

S3: Additional Data for the MeOH system 12

S4: Additional Data for the EtOAc system 14

S4.1: Details on Fragmentation Mechanism 14

S4.2: Details on Proton-Bound Dimer Chemistry 16

S4.3: Fine tuning of rate constants 19

S4.4: 2D-IMS-MS spectra 20

S5: Experimental details 21

S5.1: Shutter operating mode 21

References 21

S1: Details about the Monte-Carlo propagation

In order to describe the details of the Monte-Carlo (MC) propagation method, we will consider an example, namely protonated acetone clustering with neutral water and neutral acetone. Thus, we consider the clusters $H^+(ACE)$, $H^+(ACE)(H_2O)$, and $H^+(ACE)_2$. For this example, let us consider only the following reactions:

$$H^+(ACE) + H_2O \rightleftharpoons H^+(ACE)(H_2O) \qquad (S\,R1)$$

$$H^+(ACE) + ACE \rightleftharpoons H^+(ACE)_2 \qquad (S\,R2)$$

The rate equations then read:

$$\frac{\mathrm{d}[H^+(ACE)]}{\mathrm{d}t} = -k_{0,0}^{1,0}[H_2O][H^+(ACE)] - k_{0,0}^{0,1}[ACE][H^+(ACE)] + k_{1,0}^{0,0}[H^+(ACE)(H_2O)] + k_{0,1}^{0,0}[H^+(ACE)_2] \qquad (S1)$$

$$\frac{\mathrm{d}[H^+(ACE)(H_2O)]}{\mathrm{d}t} = +k_{0,0}^{1,0}[H_2O][H^+(ACE)] - k_{1,0}^{0,0}[H^+(ACE)(H_2O)] \qquad (S2)$$

$$\frac{\mathrm{d}[H^+(ACE)_2]}{\mathrm{d}t} = +k_{0,0}^{0,1}[ACE][H^+(ACE)] - k_{0,1}^{0,0}[H^+(ACE)_2] \qquad (S3)$$

Here, we use subscripts and superscripts (n_{H_2O}, n_{ACE}) for the rate constants, k, to denote the initial and final state of the corresponding reaction, respectively.

S1.1: Reaction Sampling via the State-Transition Matrix

As outlined in the main manuscript and described previously,[1–3] reaction sampling of the individual particles is done using the state-transition matrix. To this end, the reaction rate equations are first written in matrix form:

$$\frac{\mathrm{d}}{\mathrm{d}t}\boldsymbol{P} = \boldsymbol{k} \cdot \boldsymbol{P} \qquad (S4)$$

Where

$$\boldsymbol{P} = \begin{pmatrix} [H^+(ACE)] \\ [H^+(ACE)(H_2O)] \\ [H^+(ACE)_2] \end{pmatrix}, \qquad \boldsymbol{k} = \begin{pmatrix} -\left(k_{0,0}^{1,0}[H_2O] + k_{0,0}^{0,1}[ACE]\right) & k_{1,0}^{0,0} & k_{0,1}^{0,0} \\ +k_{0,0}^{1,0}[H_2O] & -k_{1,0}^{0,0} & 0 \\ +k_{0,0}^{0,1}[ACE] & 0 & -k_{0,1}^{0,0} \end{pmatrix} \qquad (S5)$$

For a given rate-constant matrix $\boldsymbol{k}$ and fixed time step length Δt, we obtain the state-transition matrix as:

$$\boldsymbol{\phi}(\Delta t) = \exp(\Delta t \cdot \boldsymbol{k}) \qquad (S6)$$

The matrix exponential can be calculated as a Taylor series, much like the scalar exponential:

$$\exp(\Delta t \cdot \boldsymbol{k}) = \sum_{n=0}^{\infty} \frac{\Delta t^n}{n!} \boldsymbol{k}^n \qquad (S7)$$

While this can be calculated directly, a faster and more numerically stable method is to diagonalize the rate-constant matrix first:

$$\boldsymbol{k} = \boldsymbol{TDT}^{-1} \qquad (S8)$$

where $\boldsymbol{T}$ is the transformation matrix containing the eigenvectors and $\boldsymbol{D}$ is a diagonal matrix containing the eigenvalues λ_i. Eq. (S7) then can be written as:

$$\exp(\Delta t \cdot \boldsymbol{k}) = \boldsymbol{T}\left(\sum_{n=0}^{\infty} \frac{\Delta t^n}{n!} \boldsymbol{D}^n\right)\boldsymbol{T}^{-1} \quad (S9)$$

And since $\boldsymbol{D}$ is diagonal, we can further write:

$$\boldsymbol{\phi}(\Delta t) = \exp(\Delta t \cdot \boldsymbol{k}) = \boldsymbol{T}\begin{pmatrix} e^{\lambda_1 \Delta t} & 0 & 0 \\ 0 & \ddots & 0 \\ 0 & 0 & e^{\lambda_m \Delta t} \end{pmatrix}\boldsymbol{T}^{-1} \quad (S10)$$

Note, that in order for the system to be "stable", *i.e.*, converge to an equilibrium distribution, all eigenvalues need to be real and negative.

For a background gas temperature of 300 K, solvent concentrations of 70 ppm_V H_2O and 1 ppm_V ACE, a field strength of 50 Td, and a time step of 0.5 ms, the state-transition matrix for the H^+(ACE) system is

$$\boldsymbol{\phi}(0.5\text{ ms}; 50\text{ Td}) = \begin{pmatrix} 0.962 & 0.038 & 0.000 \\ 0.037 & 0.962 & 0.000 \\ 0.001 & 0.000 & 1.000 \end{pmatrix}$$

As mentioned in the main manuscript, the state-transition matrix contains the reaction probabilities from each state to every other (column wise). This can be depicted in a diagram like the following:

Scheme S1: Reaction probabilities for the H^+(ACE) system for a time step of 10 ns at 50 Td.

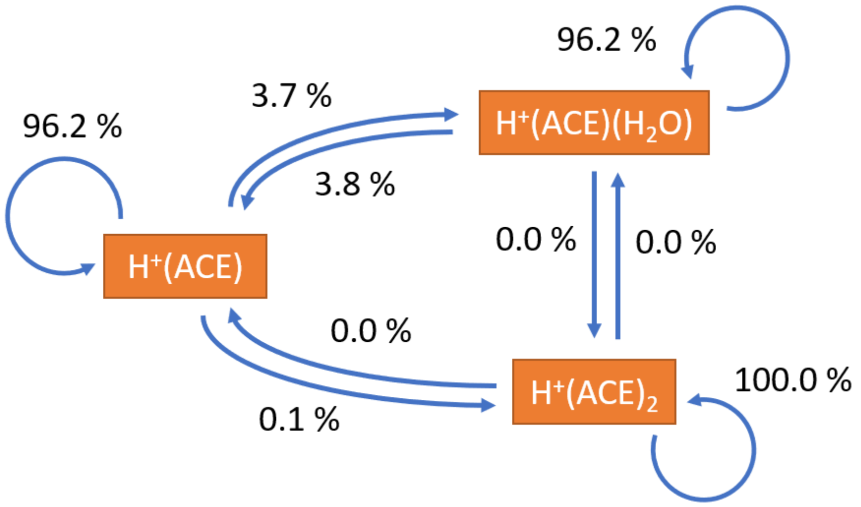

Note that the probability to not react is by far the largest. Note also, that the chosen time step of 0.5 ms is larger than what is used in the simulations (10-100 ns) to show larger numbers.

The columns of the state transition matrix represent the reaction probabilities of one species to any other species and thus sum up to 100 %. Thus, if the current identity of the particle is X, a reaction event is sampled according to the corresponding column, ϕ_{iX}. If a reaction occurs and the new species is Y, the sampling in the next time step is taken according to ϕ_{iY}.

To actually obtain the rate constants depending on the field strength, the following equations are used:[2]

$$k_{loose}^{ass}(T_{eff}) = \kappa \frac{k_B T}{h} \left(\frac{h^2}{2\pi \mu_S k_B T_{eff}^S} \right)^3 \frac{Q^*}{Q_S^r} \qquad (S11a)$$

$$k_{loose}^{diss}(T_{eff}) = \kappa \delta \frac{k_B T}{h} \frac{Q_M^{rv} Q_S^v Q^*}{Q_{MS}^{rv}} \exp\left(-\frac{\Delta\varepsilon_0}{k_B T_{eff}} \right) \qquad (S11b)$$

$$k_{tight}(T_{eff}) = \kappa \delta \frac{k_B T}{h} \frac{Q_{TS}^{rv}}{Q_M^{rv}} \exp\left(-\frac{\Delta\varepsilon_0^{\ddagger}}{k_B T_{eff}} \right) \qquad (S11c)$$

where in first approximation of 2TT:

$$T_{eff} \approx T_{bath} + \frac{M}{3k_B}(KE)^2 \qquad (S12a)$$

$$T_{eff}^S \approx T_{bath} + \frac{M_S}{3k_B}(KE)^2 \frac{m + M}{m + M_S} \qquad (S12b)$$

are the effective temperatures of the ion with respect to the bath gas and with respect to the neutral (e.g. a clustering H_2O). m is the mass of the ion, M is the mass of the bath gas, M_S is the mass of the solvent, μ_S is the reduced mass of the ion-neutral pair, K is the ion's mobility, E the applied field strength, $\Delta\varepsilon_0$ are zero-point-energy corrected threshold energies, $\kappa \in [0,1]$ is the reaction transition coefficient (usually fixed to ≈ 0.5), δ is the degeneracy of the channel, and Q are partition functions. In particular, the "loose-TS partition function" Q^* is calculated assuming either an ion-dipole (ID) or ion-induced dipole (IID) potential according to:[4]

$$Q_{ID}^*(T) = \frac{2\mu_S q \mu_D}{\hbar^2} \sum_{j,k,m} \begin{matrix} \exp\left(-\left\{ (B_{1D} - B_{2D})k^2 + B_{2D}\left\{ km + \frac{3(k-m)^2 - 3 - (2j - |m+k| + 1)^2}{8} \right\} \right\} / k_B T \right) \\ \times \int_0^1 \exp\left(-\frac{B_{2D}(2j - |m+k| + 1)^2}{2(1-G)k_B T} \right) \mathrm{d}G \end{matrix} \qquad (S13a)$$

$$Q_{IID}^*(T) = \frac{\sqrt{2\pi \mu_S^2 \alpha q^2 k_B T}}{\hbar^2} Q_S^r(T) \qquad (S13b)$$

where μ_D and α are the dipole moment and polarizability of the neutral reaction partner, respectively, and q is the charge of the ion. For the ID potential, the rotational constant along the dipole axis, B_{1D}, and the 2D rotational constant orthogonal to the dipole axis, B_{2D}, are obtained as described in Ref. [4]. Note that the description of the IID potential assumes an isotropic polarizability.

Thus, depending on the field strength, E, and the ion's mobility, K, the effective temperature can be calculated and all needed rate constants can be obtained. This makes the state-transition matrix field dependent.

S1.2: Diffusion of Particles along the Drift Length

S1.2.1: Diffusion according to Fick's second law

The diffusion of a particle in one dimension (x-direction) over time is given by Fick's second law:

$$\frac{\partial\varphi}{\partial t} = D\frac{\partial^2\phi}{\partial x^2} \tag{S14}$$

where φ is the particle concentration and D is the diffusion coefficient. Upon integration, one obtains

$$\varphi(x,t) = \frac{1}{\sqrt{4\pi Dt}}\exp\left(-\frac{x^2}{4Dt}\right) \tag{S15}$$

This is a Gaussian distribution in space with a standard deviation of $\sigma_S = \sqrt{2Dt}$ (units of length). That means, as time evolves, the distribution becomes broader, representing the diffusion in $\pm x$ direction with time. This can be used to estimate the arrival time distribution (ATD) of a non-reacting particle after its drift time, t_D. Here, however, we use this equation to determine the diffusion during each time step, Δt.

We could sample the random diffusion step d_{diff} (*cf.* Eq. (10) in the main manuscript) directly from Eq. (S15) by replacing t with Δt. This distribution has a standard deviation of $\sqrt{2D\Delta t}$ and by the law of large numbers, the distribution of the sum of N of such samples will be a Gaussian with standard deviation $\sqrt{2D \cdot N\Delta t}$ for large enough N. This then yields the correct distribution after t_D (standard deviation of $\sqrt{2Dt_D}$) as we stop the propagation exactly when $N\Delta t = t_D$. However, for any "per time step" distribution with a standard deviation of $\sqrt{2D\Delta t}$ the summed distribution will be (approach) a Gaussian with standard deviation $\sqrt{2Dt_D}$. Thus, we sample from the simple $\pm\sqrt{2D\Delta t}$ distribution since it is numerically much faster than sampling from a normal distribution. **Figure S1** shows that the distribution obtained from this 1D random-walk approach matches perfectly the expected distribution according to Fick's second law.

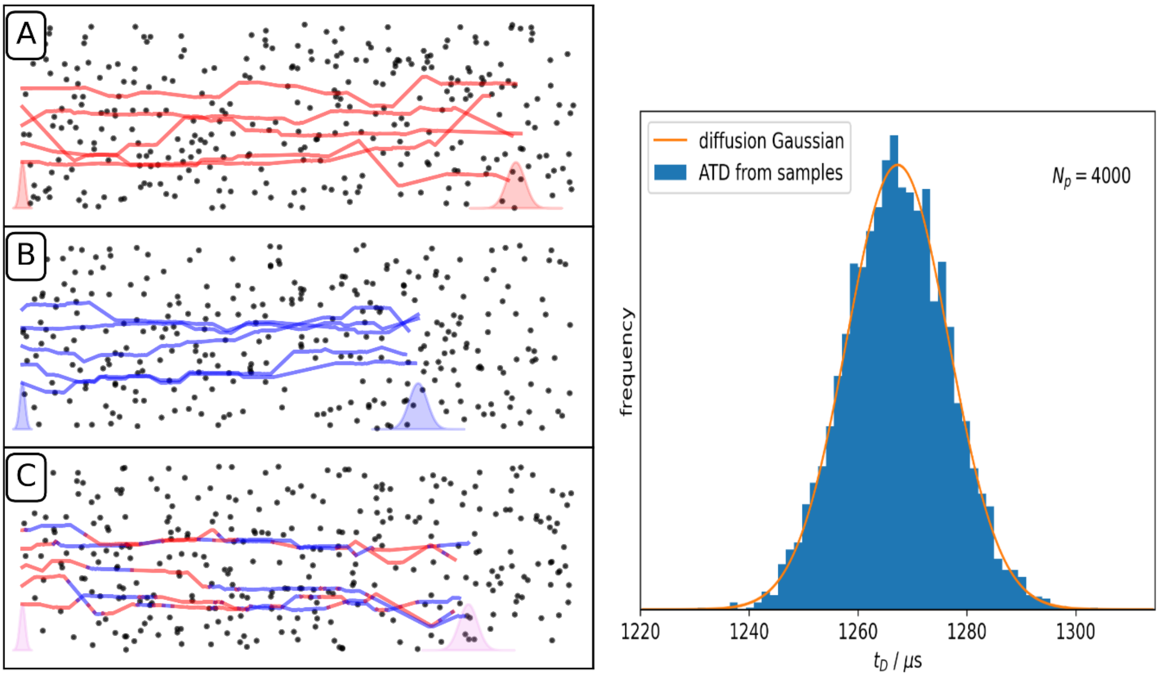

Figure S1: Left: Schematic representation of drift trajectories of two ion species (moving from left to right), marked in red and blue for (A) only red, higher v_D, (B) only blue, lower v_D, and (C)

interconverting red and blue, average v_D. Collision and reaction events are randomly sampled. Black dots represent the background gas particles. Right: Testing of random-walk diffusion. Shown is the arrival time distribution (ATD) of a non-reacting particle (4000 particles simulated) as sampled by the Monte-Carlo method presented here (incl. the random-walk diffusion) as well as the expected Gaussian distribution from Fick's second law (*i.e.*, one with standard deviation of $\sqrt{2Dt_D}$ around t_D).

S1.2.2: Diffusion coefficients at arbitrary field strengths

To execute this procedure, we need the diffusion coefficient, D, of the current particle at the specific experimental conditions (temperature, pressure) and as a function of reduced field strength. Because the mean velocity of the ions is different in the direction of the field as compared to orthogonal to the field, the diffusion coefficients in these directions also differ. This is usually described by the longitudinal diffusion coefficient, D_L, (parallel to the field) and the transverse diffusion coefficient, D_T, (orthogonal to the field). Conveniently, the generalized Einstein relations (GER) relate ion mobility to the diffusion coefficients:[5]

$$D_L = \frac{k_B T_L}{q} K(1 + K') \qquad (S16a)$$

$$D_T = \frac{k_B T_T}{q} K \qquad (S16b)$$

where K is the ion mobility coefficient (changing with the reduced field strength), $K' = \frac{d \ln K}{d \ln E}$ and $T_{L,T}$ are the (translational) ion temperatures in longitudinal and transverse direction. They are given by

$$T_{L,T} = T_{bath} + \frac{\zeta_{L,T} M}{k_B} (v_D)^2 \qquad (S17)$$

with M being the mass of the bath gas particles and

$$\zeta_L = \frac{5m - (2m - M)A^*}{5m + 3MA^*} \quad , \quad \zeta_T = \frac{(m + M)A^*}{5m + 3MA^*} \qquad (S18)$$

Here, $A^* = \Omega^{(2,2)}(T_{eff})/\Omega^{(1,1)}(T_{eff})$ and m is the ion's mass. Note that $T_L \geq T_{eff} \geq T_T$. Thus, we obtain the diffusion coefficients of each species directly from the mobility data at the current reduced field strength.

S1.2.3: Diffusion broadening for varying fields

As mentioned before, the diffusion coefficient depends on the field strength. This is not only due to the field dependency of the mobility coefficient, K, but also because $T_{L,T}$ depend on the field. Thus, it is of interest to investigate, how the peak width, caused by diffusion, evolves with field strength. Generally, we need to distinguish between the peak width in space, σ_s, and in time, σ_t, whereby the former is defined by Fick's second law and the latter is what is observed as the width of the ATD (in case of an infinitesimally narrow initial distribution). The two are connected via:[6]

$$\frac{\sigma_S}{L} = \frac{\sigma_t}{t_D} \qquad (S19)$$

By inserting Eq. (S16a) into $\sigma_s = \sqrt{2D_L t_D}$, and further using $t_D = L/(KE)$, we arrive at

$$\sigma_s = \sqrt{\frac{2L}{q}} \sqrt{k_B T_L} \sqrt{\frac{1 + K'}{E}} \qquad (S20a)$$

$$\sigma_t = \sqrt{\frac{2L}{q}}\sqrt{\frac{k_B T_L}{K^2}}\sqrt{\frac{1+K'}{E^3}} \qquad (S20b)$$

However, the quantities T_L and K' are not always known. Hence approximations are needed.

In the low-field limit, $T_L \rightarrow T_{bath}$ as per Eq. (S17), $K \rightarrow K_{lf}$, and $K' \rightarrow 0$ and we can write:

$$\sigma_s^{lf} = \sqrt{\frac{2L}{q}}\sqrt{k_B T_{bath}} \cdot E^{-1/2} \qquad (S21a)$$

$$\sigma_t^{lf} = \sqrt{\frac{2L}{q}}\sqrt{\frac{k_B T_{bath}}{K_{lf}^2}} \cdot E^{-3/2} \qquad (S21b)$$

On the other hand, in the high-field limit, $k_B T_L \rightarrow \zeta_L M (KE)^2$, as this term becomes dominant over T_{bath} in Eq. (S17). Still assuming $K' \approx 0$, we can write:

$$\sigma_s^{hf} = \sqrt{\frac{2L}{q}}\sqrt{\zeta_L M K^2} \cdot E^{+1/2} \qquad (S22a)$$

$$\sigma_t^{hf} = \sqrt{\frac{2L}{q}}\sqrt{\zeta_L M} \cdot E^{-1/2} \qquad (S22b)$$

We can further approximate ζ_L by setting $A^* = 1$ in Eq. (S18) and if no high-field mobilities are available, we can even use K_{lf} instead of K in Eq. (S22a). This makes calculations of $\sigma_{s/t}^{lf/hf}$ possible from very little data.

Because of the two terms contributing to T_L (T_{bath} and the field dependent term), it is a fairly good approximation to use

$$\sigma_{s/t}^{approx} = \sqrt{\left(\sigma_{s/t}^{lf}\right)^2 + \left(\sigma_{s/t}^{hf}\right)^2} \qquad (S23)$$

to approximate the spatial/temporal peak width over the entire range of field strength. This approximation is tested for $H^+(ACE)$ against Eq. (S20) in **Figure S2**. As can be seen, the approximation presented in Eq. (S19), is very good over the whole range of field strengths, while the two limiting approximations (low-field and high-field limit), are only good approximations at their respective limits. Specifically, the low-field approximation, which has been used before to estimate the amount of diffusion,[6,7] predicts much lower standard deviations than the approximation of Eq. (S23).

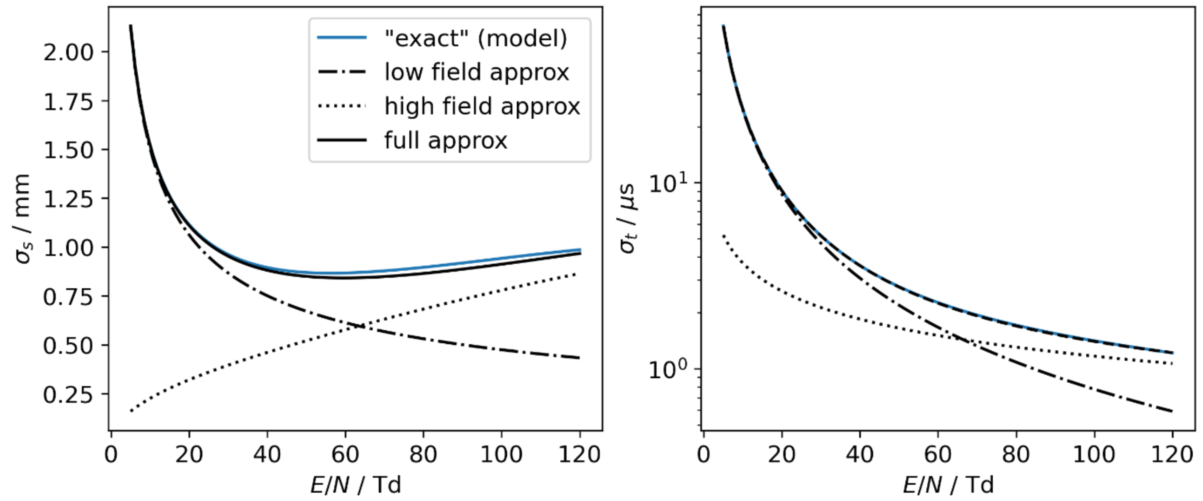

Figure S2: Spatial and temporal standard deviations of the ion distribution caused by diffusion determined by Eq. (S20) ("exact"), and different levels of approximations for $H^+(ACE)$. These use $A^* = 1, K' = 0$ but full, field-dependent mobility data K. The drift length is 150 mm.

In terms of the diffusion contribution to the peak width, $w_{0.5}^{diff}$, we use the temporal standard deviation, σ_t, and the fact that $w_{0.5} = \sqrt{8\ln(2)}\,\sigma_t$ to arrive at:

$$w_{0.5}^{lf} = \sqrt{\frac{16\ln(2)\,L}{q}}\sqrt{\frac{k_B T_{bath}}{K_{lf}^2}} \cdot E^{-3/2} \qquad (S24a)$$

$$w_{0.5}^{hf} = \sqrt{\frac{16\ln(2)\,L}{q}}\sqrt{\zeta_L M} \cdot E^{-1/2} \qquad (S24b)$$

For the relative peak width, $w_{0.5}^{diff}/t_D$, using $t_D = L/KE$, we arrive at

$$w_{0.5}^{lf}/t_D = \sqrt{\frac{16\ln(2)}{qL}}\sqrt{k_B T_{bath}} \cdot E^{-1/2} \qquad (S25a)$$

$$w_{0.5}^{hf}/t_D = \sqrt{\frac{16\ln(2)}{qL}}\sqrt{\zeta_L M}\,K \cdot E^{+1/2} \qquad (S25b)$$

This yields the dependencies of $w_{0.5}^{diff}/t_D$ mentioned in the main text. Importantly, in the high field limit, the relative peak width (i.e., its diffusion contribution) actually increases with field strength due to the increased diffusion coefficient.

S1.3: Comparison to Markov-chain Method

To validate the performance of the new MC propagation methodology, it is warranted to compare it to the previously used Markov-chain method. To this end, we expand the previous example of H^+(ACE) to higher cluster numbers (including mixed clusters) of the general type [H^+(ACE)+*n*(H_2O)+*m*(ACE)] or (*n*,*m*) for short. It was previously shown that the Markov-chain propagation is able to reproduce the cluster size distribution of this system as measured by the HiKE-IMS over a wide range of reduced field strengths.[3] In **Figure S3** we compare the temporal evolution of this system as predicted by the Markov-chain as well as the MC model at two different reduced field strengths (40 and 70 Td) in the HiKE-IMS.

The background gas temperature is 300 K, and we assume 70 ppm$_V$ H_2O and 1 ppm$_V$ of neutral ACE. For both field strengths it can be seen that the initial cluster distribution is changing over the course of the drift time and that the two methods (Markov-chain and MC) agree very well. As the Markov-chain model is an analytical solution to the rate equations, the predicted concentrations evolve smoothly. In contrast, the MC method simulates discrete particles and random sampling, which leads to small fluctuations in the shown traces. These fluctuations become smaller as the number of simulated particles is increased and for the 1000 particles used here, we already see rather small fluctuations. This shows that the MC method is able to produce the same data as the previously validated Markov-chain module.

It should be noted that the MC method takes significantly more computing time as compared to the Markov-chain approach. The Markov-chain method can be thought of the MC method with only one, average particle. Hence, to a first approximation, the MC method take N_p times more computing time than a comparable Markov-chain calculation. In other words, both methods scale linearly with the number of time steps ($\mathcal{O}(N_{\Delta t})$, but the MC method additionally scales linearly with the number of simulated particles ($\mathcal{O}(N_p)$). While we did not find this to be an issue for this work (N_p on the order of 10^3 and Δt on the order of 10^{-7} s completing in a couple of seconds on a laptop), simulations with very large particle numbers or very small time steps (e.g., to sample a high frequency oscillating field as in DMS/FAIMS) could make parallelization of the code necessary in order to keep computing time short.

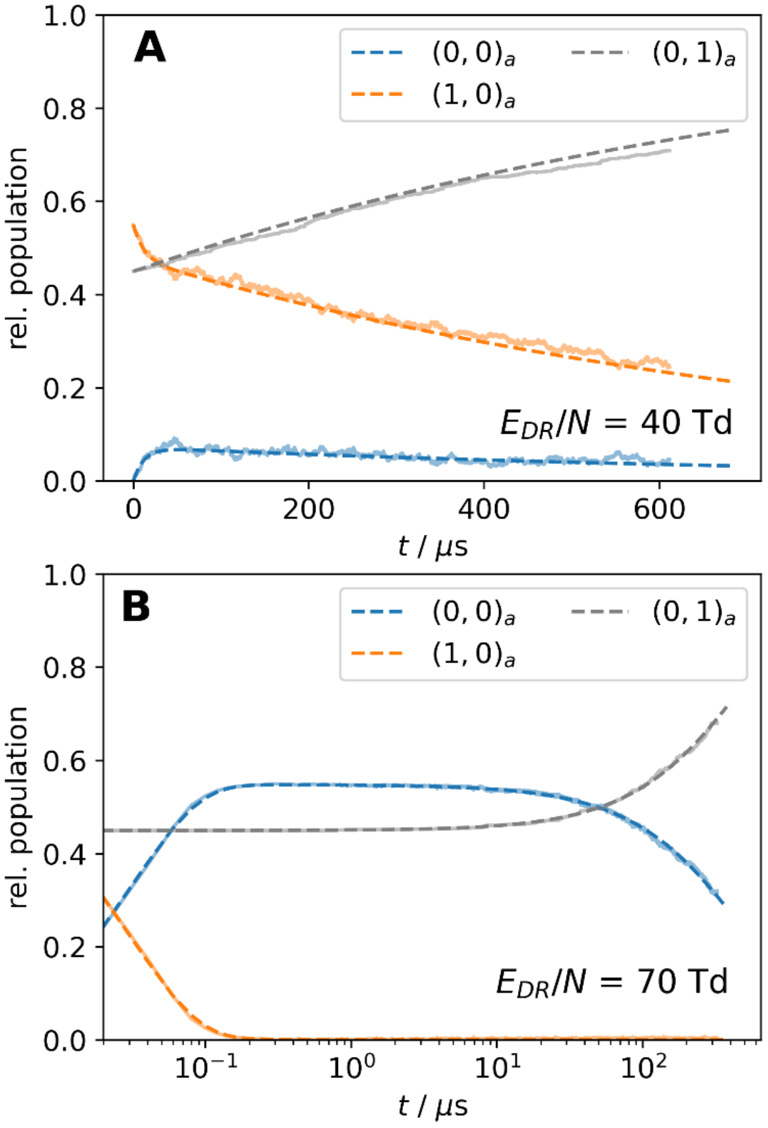

Figure S3: Cluster size population evolution as evaluated by the Markov-chain method (dashed lines) and the Monte-Carlo method (transparent solid lines, 1000 particles) for the [H^+(ACE)+n(H_2O)+m(ACE)] system, *viz*. (n,m) in the figure legend, for (A) 40 Td (Δt=50 ns) and (B) 70 Td (Δt=20 ns). Note the logarithmic time axis for panel (B).

S2: Additional Data for 2,6-D*t*BP

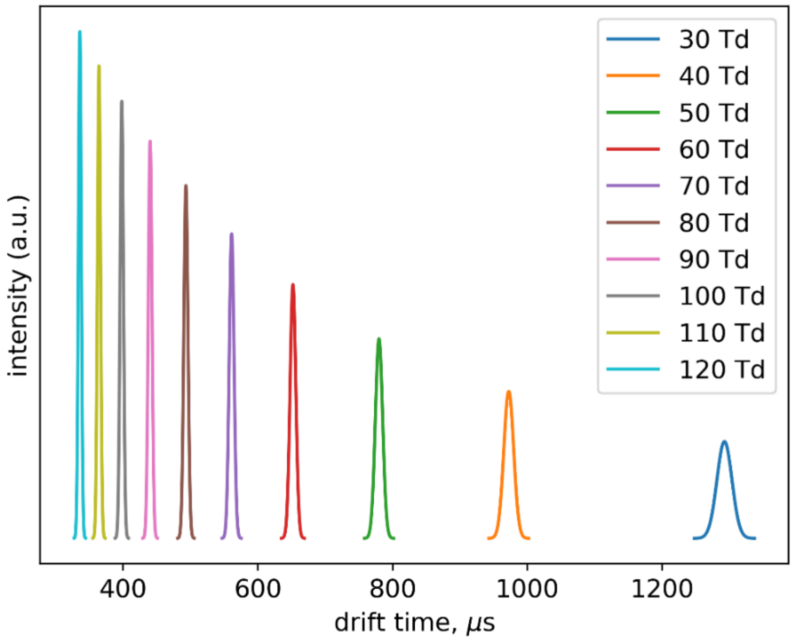

Figure S4: Gaussian fits (normalized) to experimentally measured ATDs for 2,6-D*t*BP over a range of reduced field strengths. Data recorded at 14.7 mbar and 80 °C using the stand-alone HiKE-IMS.

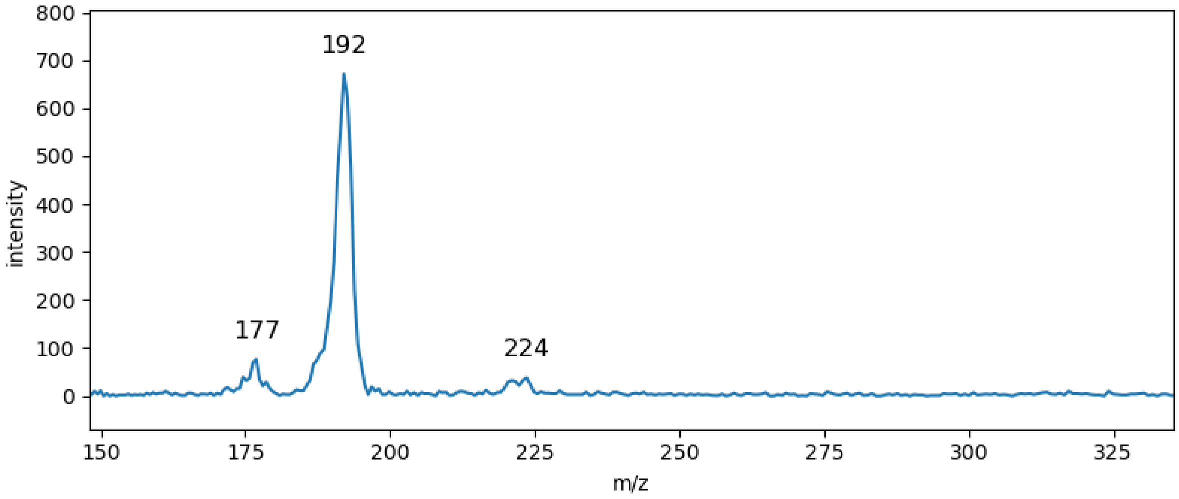

Figure S5: Mass spectrum of [2,6-D*t*BP+H]$^+$ (m/z 192) recorded with the HiKE-IMS-MS at a reduced field strength of 30 Td in the reaction and drift region. Note the lack of [2,6-D*t*BP+H+H_2O]$^+$ (m/z 210).

Based on the calculated binding energy of H_2O to 2,6-D*t*BP ($\Delta\varepsilon_0 = 41.3\ \mathrm{kJ/mol}$) and the assumed water concentration of 70 ppm$_V$, the ratio of free to hydrated analyte is 10^7:1 under the given temperature and pressure conditions. Thus, the clustering with water can be neglected for 2,6-D*t*BP.

S3: Additional Data for the MeOH system

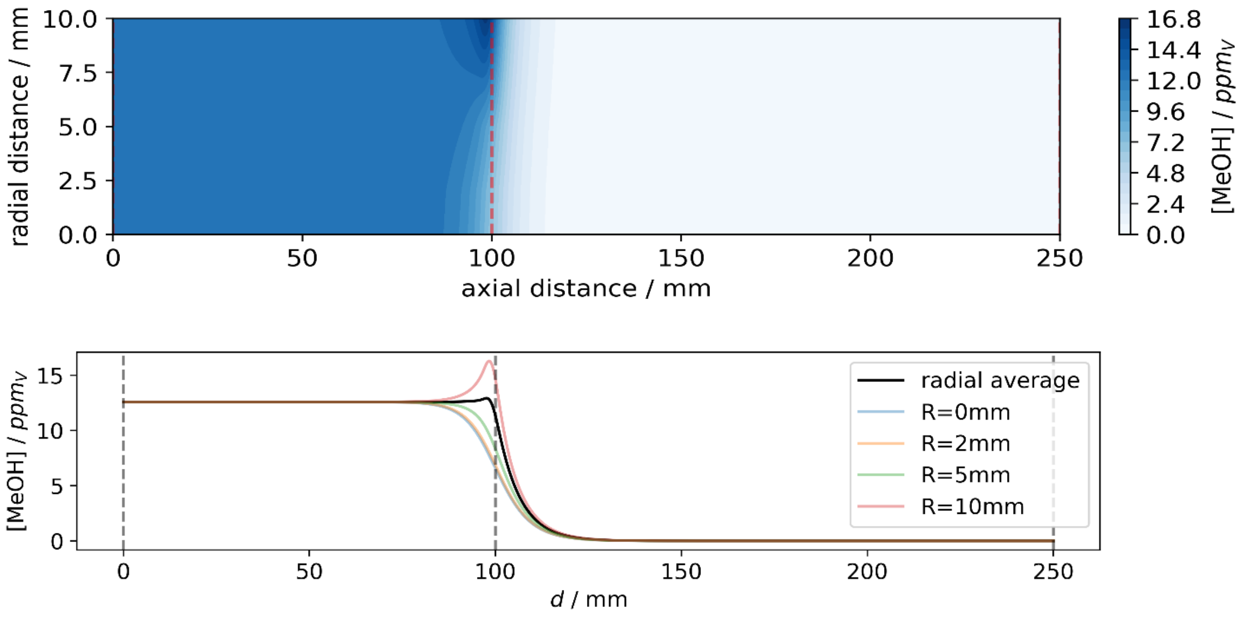

Figure S6: Neutral MeOH concentration in the reaction region (RR, 0 – 100 mm) and drift region (DR, 100 – 250 mm). Top: contour plot of MeOH concentration over the length and radius of the instrument. Bottom: Concentration profiles over the length of the instrument at different distances from the center as well as radially averaged concentration profile (black line). The latter can be approximated in the DR by $11.7\ ppm_V \times exp\left(-165.8(d-0.1m)\right)$, where d is the distance in meters (from 0.1 – 0.25 m). Modeling done using COMSOL.

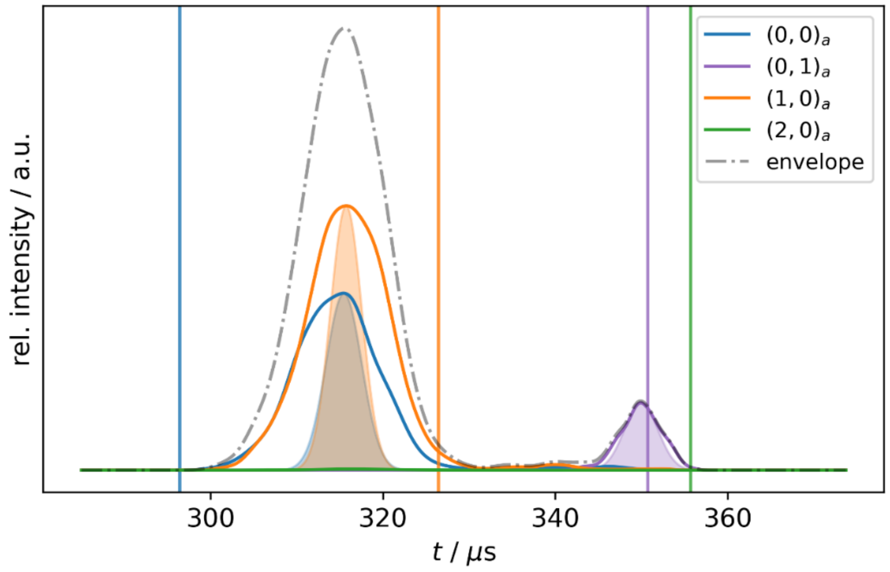

Figure S7: Arrival time distributions of the different cluster species in the [$MeOH_2^+$+n(H_2O)+m(MeOH)], *viz*. (*n*,*m*) in the figure legend, system at 70 Td as calculated via the MC framework (4000 particles). The shaded areas correspond to the peak widths expected from diffusion according to Fick's second law, the colored vertical lines correspond to the expected arrival times of the individual species if chemically isolated. First, it can be seen that the ATD of the hydrates is shifted as compared to the bare ion. Further, the actual peak widths are increased as compared to the diffusion peak width due to the cluster chemistry occurring during the ion transit.

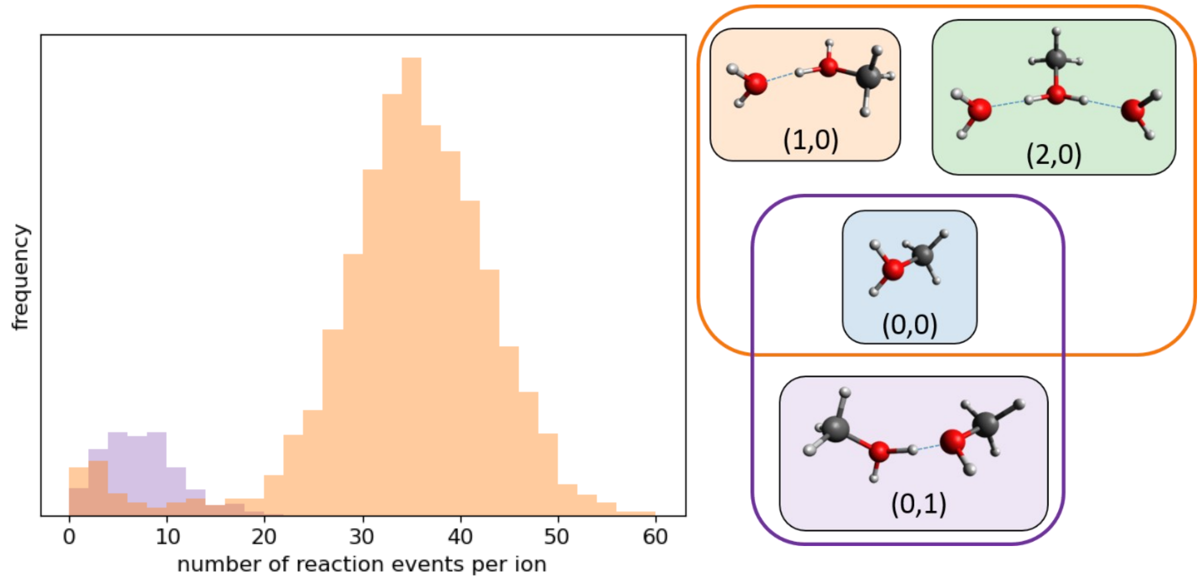

Figure S8: Number of reaction events occurring per ion associated with the bare ion and its hydrates (orange) and the dimer (purple). Data obtained at 70 Td through the MC framework.

S4: Additional Data for the EtOAc system

S4.1: Details on Fragmentation Mechanism

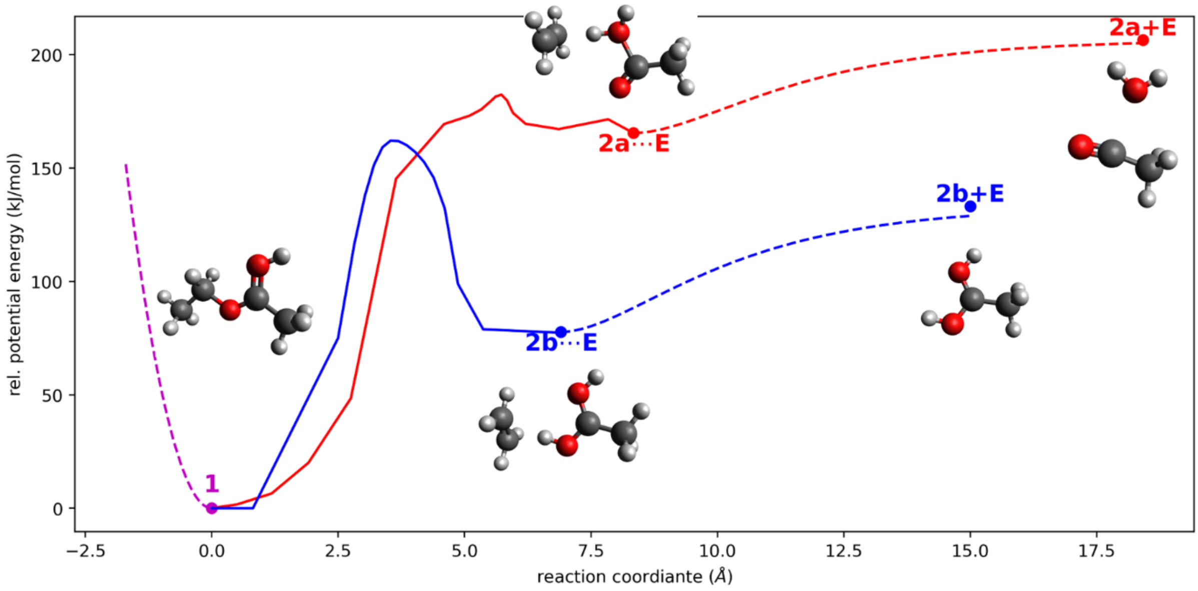

Figure S9: Minimum Energy Paths (MEPs) as determined by the NEB method for the fragmentation of **1** (protonated EtOAc) to **2a** and **2b** (loss of **E** = C_2H_4). Both channels show a tight TS towards a loosely bound complex of the fragment with the ethene, which then dissociates via a loose TS towards the separated products. The latter path is approximated by a Morse potential here (dashed lines). Note that, although the barriers to form the dissociative complex are of similar height, the reverse reaction is much more favorable for **2a…E**. This results in a much lower overall dissociation rate for the **a** channel as compared to the **b** channel. Only at high reduced field strengths, when the overall threshold to **2a+E** is reached, this channel opens up.

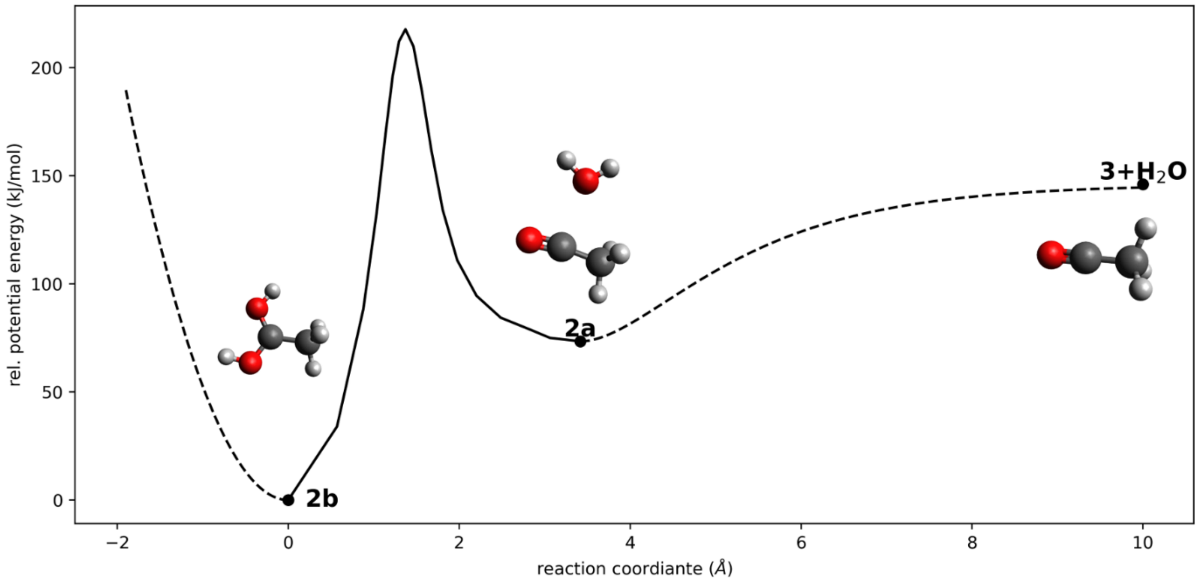

Figure S10: Minimum Energy Paths (MEPs) as determined by the NEB method for the interconversion between **2a** and **2b** as well as the fragmentation of **2a** into **3** (loss of H_2O). While the interconversion proceeds through a tight TS, the loss of H_2O proceeds through a loose TS as the H_2O fragment is already only loosely bound in **2a**. The latter path is approximated by a Morse potential here (dashed lines).

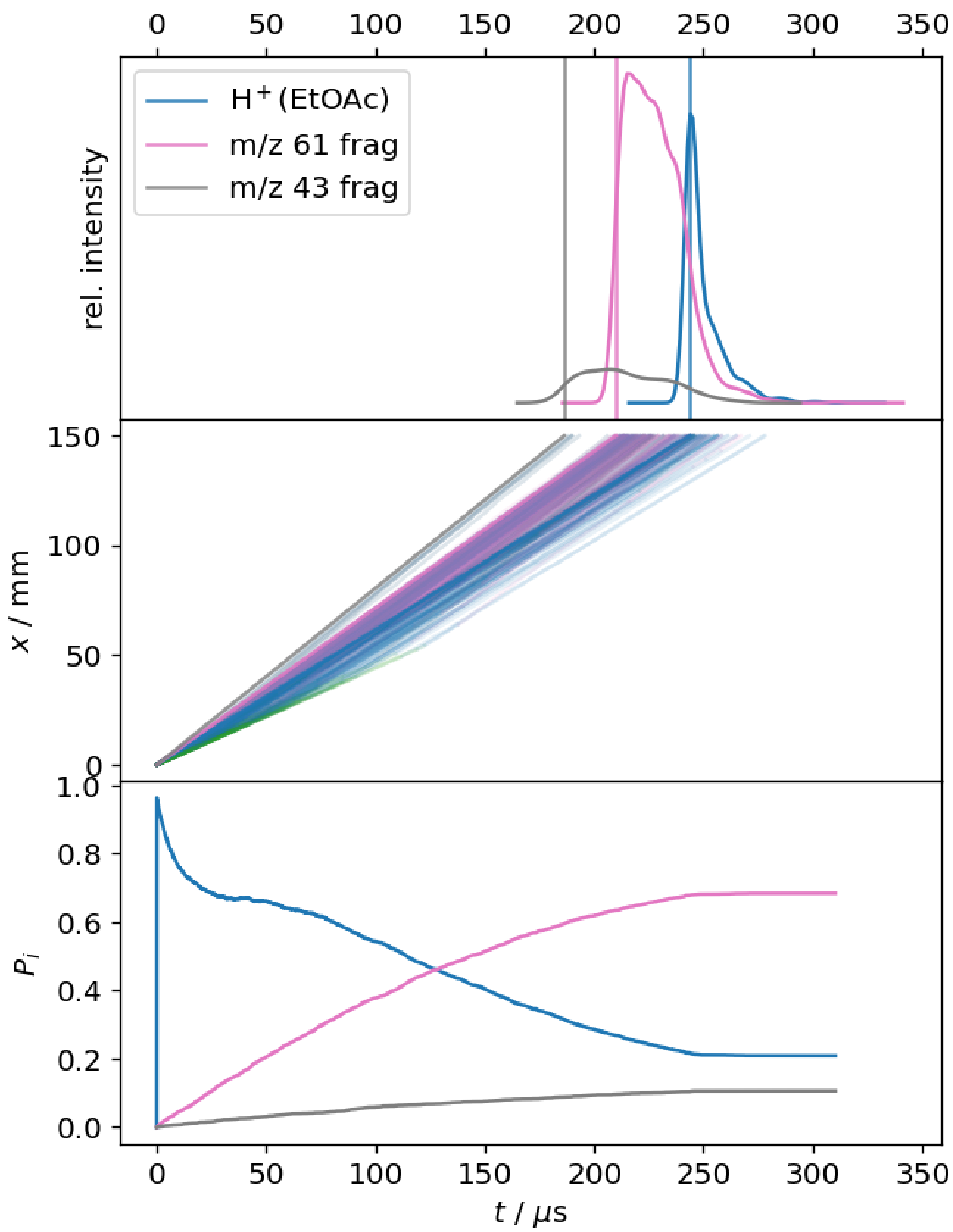

Figure S11: ATD, ion trajectories and relative populations at 110 Td showing the somewhat slow fragmentation of the parent ion and the resulting tailing of the fragment ATD. In particular, when ions start as parent ion but then fragment, their drift velocity increases (larger slope in the center plot), yielding an arrival time between the one of the parent and fragment. As the reaction proceeds through a first order kinetic, more fragmentation occurs early on, producing the typical tailing behavior. The situation here is further complicated through the influence of the proton bound dimer, creating additional tailing of the parent ion ATD towards longer drift times (see next section). The dimer chemistry is also responsible for the unusual decay of the bare ion seen in the lower panel.

S4.2: Details on Proton-Bound Dimer Chemistry

As we saw in our previous publication[3] on protonated acetone in the HiKE-IMS-MS, the proton-bound dimer (PBD) of the analyte, $H^+(EtOAc)_2$, plays an important role in the ion chemistry at low and medium reduced field strengths. For one, the PBD can form already in the reaction region and due to its high stability (as compared to hydrates of the analyte) can be detected over a wide range of reduced field strengths (see Figure 5 of the main manuscript). Moreover, if significant amounts of neutral EtOAc diffuse into the drift region, the PBD can be formed during the ion mobility separation. This can occur either by clustering of the protonated bare ion with the neutral analyte:

$$H^+(EtOAc) + EtOAc \rightleftharpoons H^+(EtOAc)_2 \qquad (S\ R3)$$

or through a ligand switching mechanism, whereby the water adduct in the monohydrate is replaced by the neutral analyte:

$$H^+(EtOAc)(H_2O) + EtOAc \rightleftharpoons H^+(EtOAc)_2 + H_2O \qquad (S\ R4)$$

The potential energy surface of the latter mechanism is shown in **Figure S*12***. Note that the mechanism proceeds through a mixed cluster of the form $H^+(EtOAc)_2(H_2O)$. Further note that replacing the H_2O ligand with a EtOAc ligand is energetically favorable.

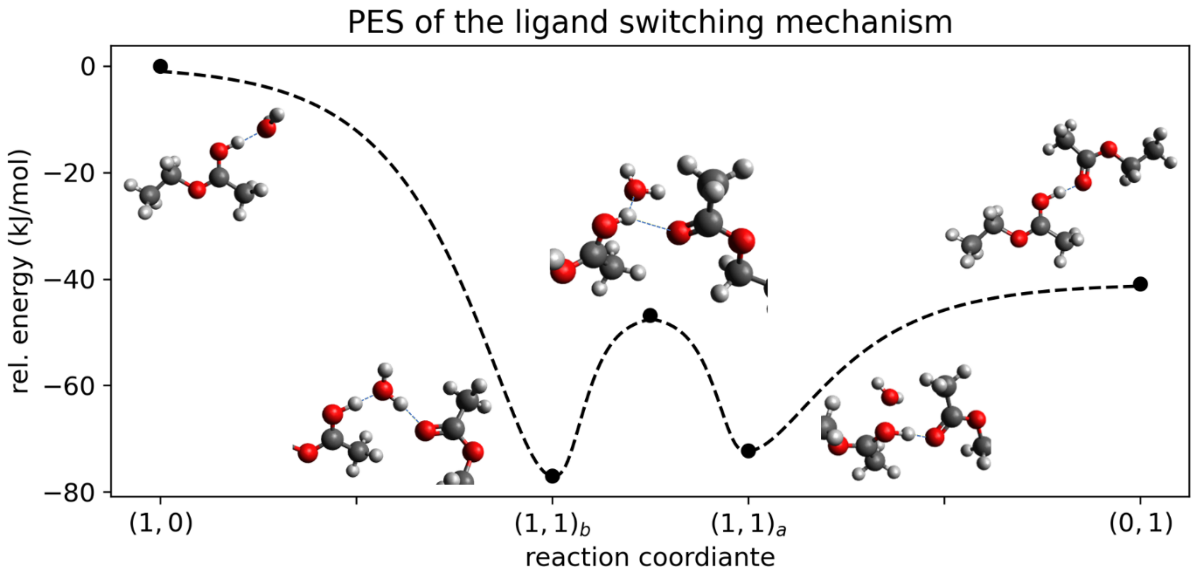

Figure S12: Potential energy surface (PES) of the ligand switching mechanism to replace the H_2O ligand bound to the protonated EtOAc with a neutral EtOAc ligand to form the proton bound dimer. Energies are zero-point energy corrected electronic energies. Dashed lines schematically show the PES between the stationary points.

As we saw previously, the ligand switching mechanism is responsible for a significant amount of PBD observed at low reduced field strengths. The COMSOL simulations conducted for MeOH (cf. **Figure S6**) suggest an inhomogeneous neutral concentration profile caused by diffusion from the reaction region. The profile can be approximated by a decaying exponential with respect to distance. As the initial concentration of the PBD and the concentration profile are unknown, we estimated these parameters based on the MeOH COMSOL simulations and comparison to the experimentally observed populations. This yielded an initial dimer population of 25 % and a concentration profile described by $[EtOAc] = 30\ \mathrm{ppm_V} \times \exp\,(-120\ \mathrm{m}^{-1} \cdot d)$, where d is the distance in the drift tube in meters. It should be noted that the results are not very sensitive to these parameters.

The concentration profile and the effect of the ligand switching mechanism are shown in **Figure S13** for a reduced field strength of 30 Td. As can be seen, the neutral EtOAc concentration quickly decays over the drift length. As a result, additional PBD is only formed in the first few cm of the drift tube, after which the PBD concentration remains constant (no further ligand switching as no neutral EtOAc is left).

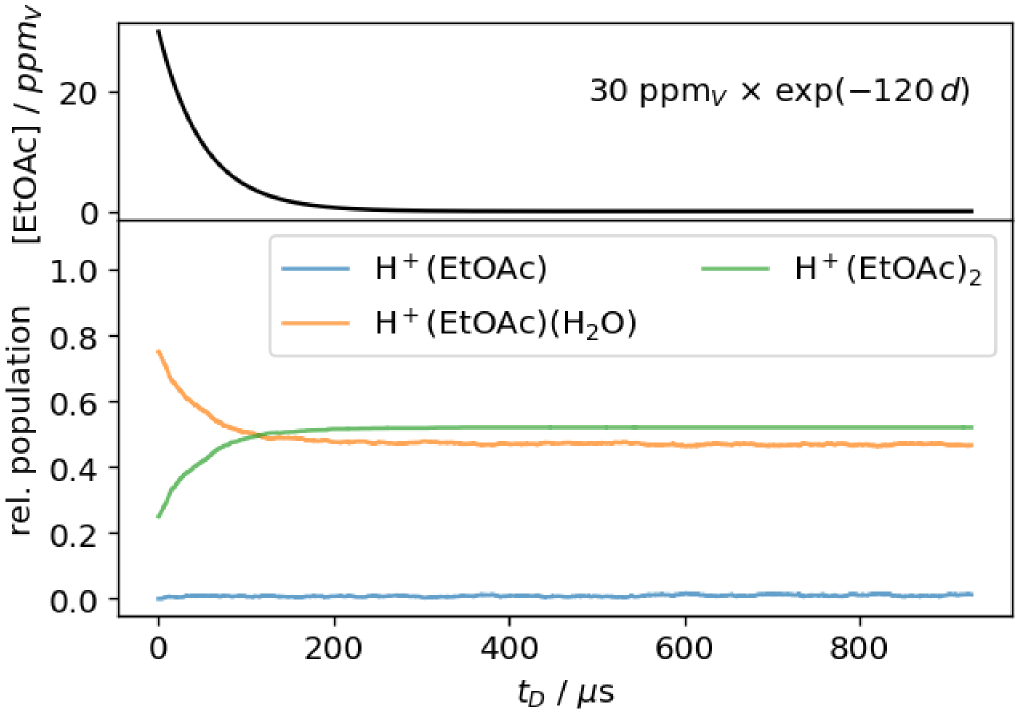

Figure S13: Neutral EtOAc concentration profile (expressed over time instead of distance for better visualization) and resulting ion populations (as driven by the ligand switching mechanism) at 30 Td.

At higher reduced field strengths, the monohydrate, $H^+(EtOAc)(H_2O)$ quickly dissociates. Thus, additional dimer formation occurs through direct reaction of the bare ion and the neutral analyte (Reaction S R3). While the reverse rate (dissociation of the PBD) is constant throughout the drift tube, the forward rate (formation of the PBD) depends on the solvent concentration at the current position of the ion cloud. As a result, at certain reduced field strength, we first observe additional dimer formation ($k_{ass}[EtOAc] > k_{diss}$), followed by predominant dissociation ($k_{ass}[EtOAc] < k_{diss}$). This can be seen in **Figure S14** (lower panel).

This dynamic behavior gives rise to the unusual ATD where both a tailing from the monomer to the dimer peak (caused by dimer fragmentation) and a fronting from the dimer to the monomer peak (caused by dimer formation) is observed.

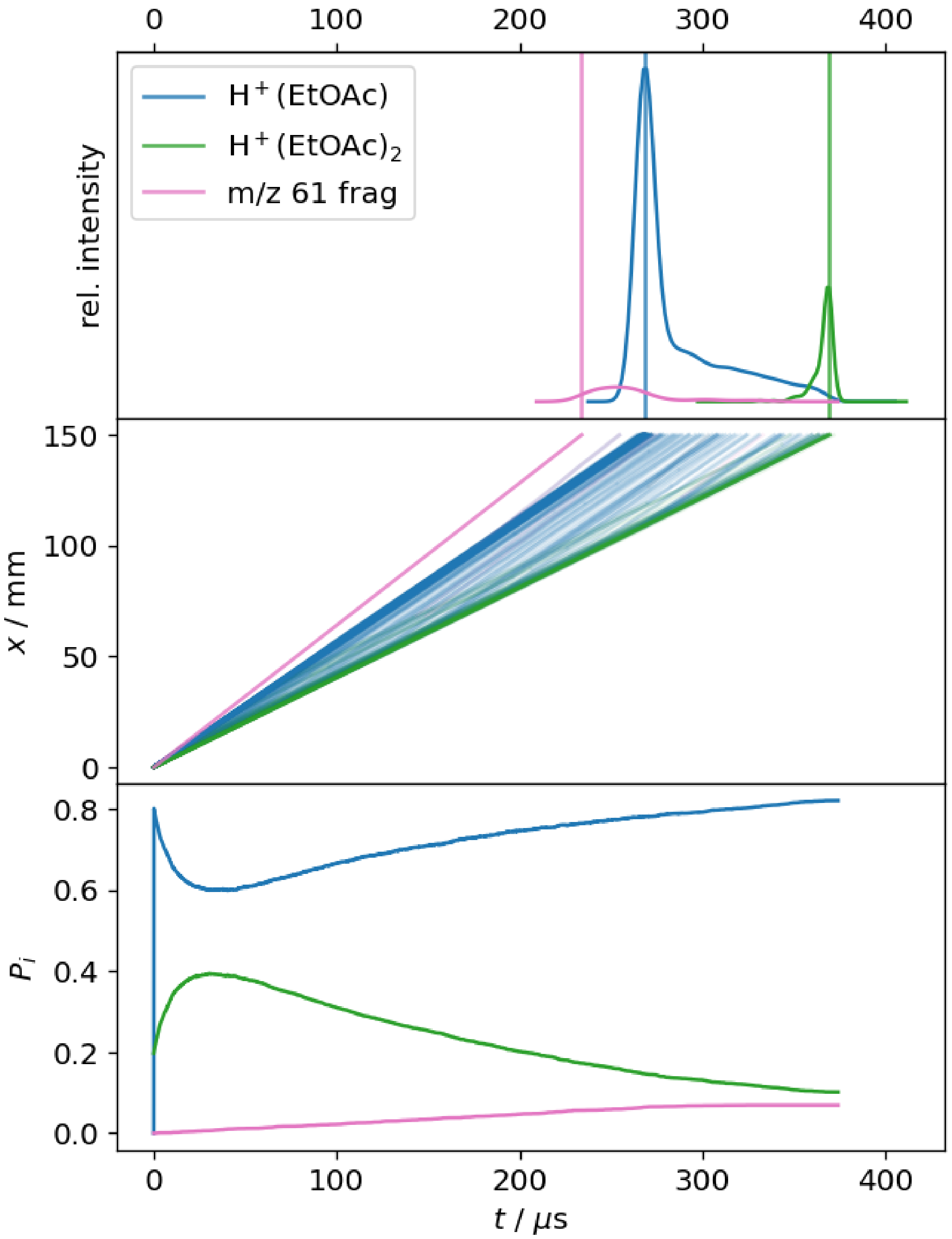

Figure S14: ATD, ion trajectories and relative populations at 100 Td showing dimer formation and subsequent dissociation and the resulting ATD profile (tailing and fronting).

S4.3: Fine tuning of rate constants

A number of uncertainties are introduced throughout the described workflow. First, the DFT calculations to determine the energies and structures of the species involved can introduces errors. We suspect that especially accurate thermochemistry is difficult to predict.[8] Further, MobCal-MPI 2.0 introduces errors in the mobility and thus the effective temperatures of the ions. Deviations in the drift times when compared to experimental data can be seen in the main manuscript. Combining these uncertainties, the determination of reaction rates is quite difficult. We noticed in the past that fast equilibria (e.g. water clustering) are less prone to errors as here, mostly the ratio of the rate constants is important, determining the position of the equilibrium.[3] Consequently, there might be large error cancellation when determining the ratio of reaction rates. Here, however, absolute rates are important as the described fragmentations do not exhibit a reverse reaction.

When comparing predicted and measured population plots as a function of reduced field strength, we noticed that the described fragmentation reactions occur too soon (too low E/N values), or in other words, too fast (see **Figure S15A**). Additional to the above-mentioned uncertainties, this might be because at very high reduced field strengths (> 100 Td), the actual fragmentation rate might be smaller than its high-pressure limiting rate, as described through the Lindemann-Hinshelwood theory. We thus decided to globally scale all rates corresponding to the fragmentation mechanism by a factor of 1/5. Rates corresponding to the formation of loosely bound clusters (proton-bound dimer, water cluster), are unaffected. This gave satisfactory results as shown in **Figure S15B** and **Figure 5** of the main manuscript. Note that scaling all rates corresponding to the fragmentation (*e.g.*, the tight TS from **2b** to **2a**, cf. **Figure S*10***) does not affect the equilibrium distribution, as this is given by the ratio of the rates, cancelling any correction factor.

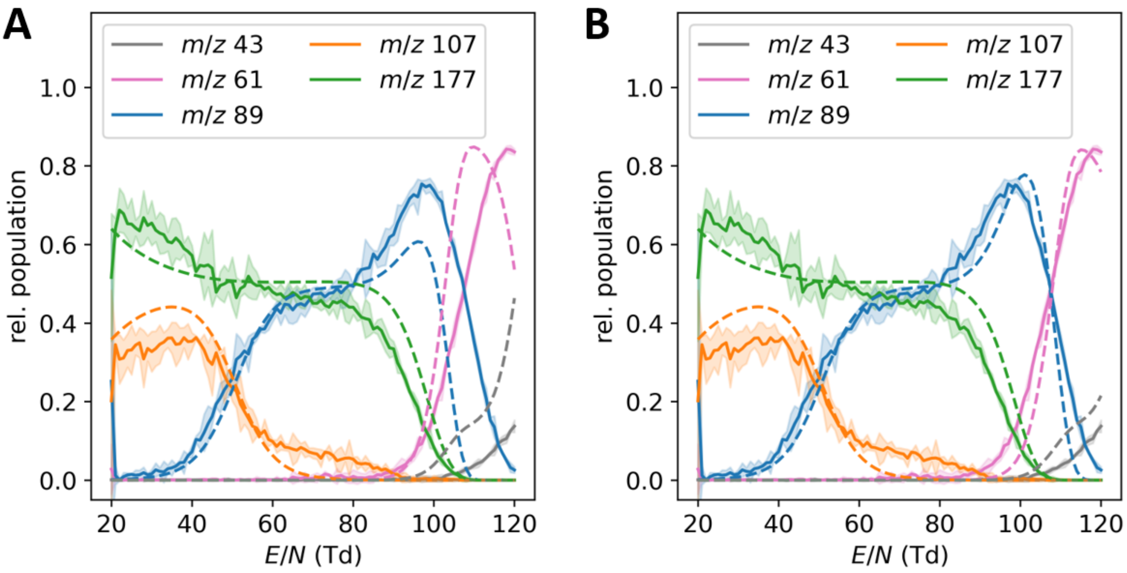

Figure S15: Comparison of population vs E/N plots using uncorrected reaction rates (A) and corrected reaction rates, *i.e.*, fragmentation rates scaled by 1/5 (B). Note that the fragmentation of the parent ion towards the m/z 61 fragment occurs at lower E/N when compared to the experimental data.

While manual tuning of reaction rates in a first-principles workflow is unideal, we decided to do so for better comparison of the arrival time distributions. If the population plots (calculated vs. measured) are slightly shifted with respect to each other, it is difficult to compare the ATDs at a common reduced field strength. Current efforts are being directed towards reducing the uncertainties associated with the reaction rates.

S4.4: 2D-IMS-MS spectra

Mass-resolved ATDs were extracted for the masses of the know species in the EtOAc system from 2D-IMS-MS data shown in **Figure *S16***. For each reduced field strength, three full 2D-IMS-MS spectra were recorded and the mass-resolved ATD extractions averaged.

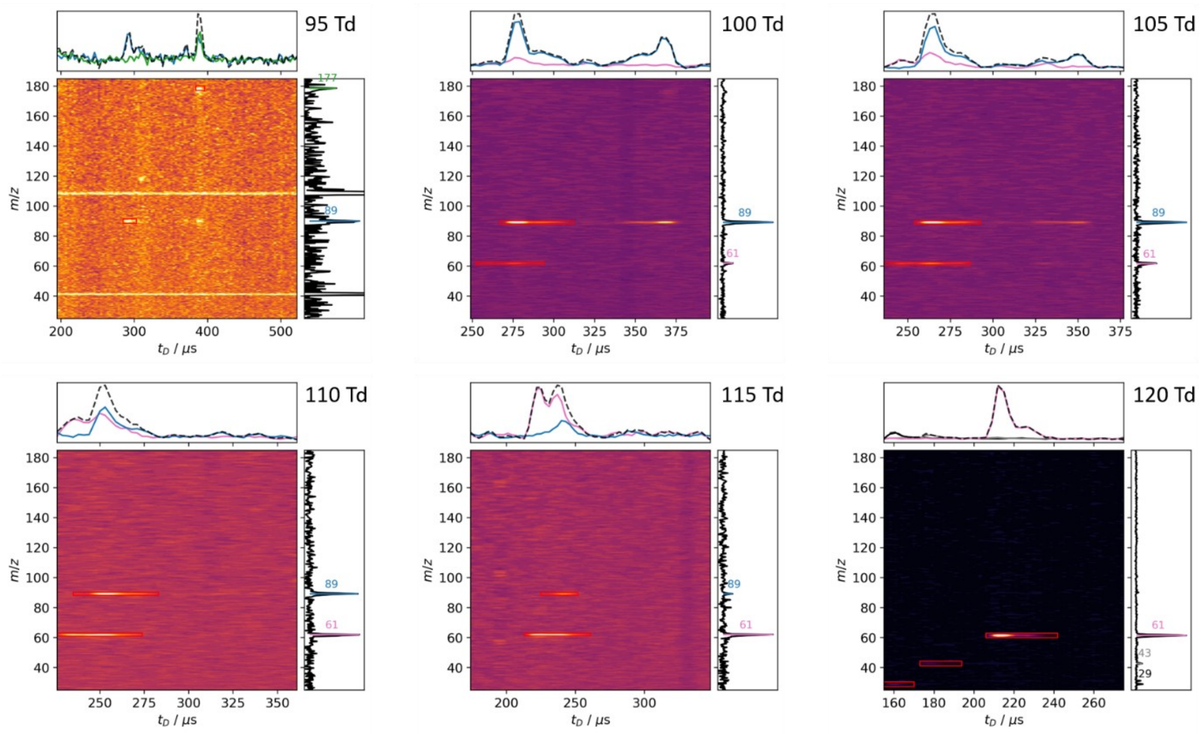

Figure S16: 2D-IMS-MS spectra for the EtOAc system at reduced field strengths between 95 – 120 Td. The most dominant species are the bare ion (m/z 89) and the first fragment (m/z 61). Minor contributions of the proton-bound dimer (m/z 177) and the second fragment (m/z 43) are visible at the lowest and highest reduced field strength, respectively.

S5: Experimental details

S5.1: Shutter operating mode

To come up for the limitations of coupling HiKE-IMS with TOF-MS due to similar time scales of the ion mobility separation and the time of flight, three operational modes for the HiKE-IMS-MS have been used in earlier work: 1. *IMS-Only Mode*, where the ion shutter 1 (prior to the drift region) is opened for a short time to inject the ion population into the drift region and the second grid of ion shutter 2 (after the drift region) is connected to a transimpedance amplifier and used to record the ion mobility spectra. 2. *Continuous-Ion-Flow Mode*, where the potentials both ion shutter 1 and ion shutter 2 are opened continuously to transfer all ions simultaneously and continuously from the reaction region into the MS. In this mode, the drift region just acts as an ion guide. 3. *Selected-Mobility Mode*, where ion shutter 1 is opened in the same way is in IMS-Only mode, but ion shutter 2 is opened with a specific time delay after ion shutter 1. In this operating mode, the drift region acts as a mobility filter and ion shutter 2 correspondingly selects only specific sections of the ion mobility spectrum for analysis in the MS.

Hence, neither *IMS-Only Mode*, nor *Continuous-Ion-Flow Mode* are feasible to record arrival time distributions for each m/z, since they can only record either ion mobility or mass spectra. The *Selected-Mobility Mode* instead is used to identify the ion species underlying specific peaks in the ion mobility spectrum. In order not to have to manually select sections from the ion mobility spectrum for further mass analysis and to be able to investigate a larger mobility range in the MS, the *2D-IMS-MS mode* as introduced in previous work[9] is used. This mode records a mass spectrum for each interval of the arrival time distributions and can thus confidently assign peaks in the ion mobility spectrum to the associated ion species. In addition, this operating mode allows to unfold peaks in the ion mobility spectrum consisting of multiple ion species.

References

(1) Alexander Haack; Justine R. Bissonnette; Christian Ieritano; W. Scott Hopkins. Improved First-Principles Model of Differential Mobility Using Higher Order Two-Temperature Theory. *J. Am. Soc. Mass Spectrom.* **2022**, *33*, 535–547.

(2) Alexander Haack; W. Scott Hopkins. Kinetics in DMS: Modeling Clustering and Declustering Reactions. *J. Am. Soc. Mass Spectrom.* **2022**, *33*, 2250–2262.

(3) Haack, A.; Schaefer, C.; Zimmermann, S.; Hopkins, W. S. Validation of Field-Dependent Ion-Solvent Cluster Modeling via Direct Measurement of Cluster Size Distributions. *J. Am. Soc. Mass Spectrom.* **2023**, *34*, 1035–1046.

(4) Troe, J. Statistical adiabatic channel model for ion–molecule capture processes. II. Analytical treatment of ion–dipole capture. *J. Chem. Phys.* **1996**, *105*, 6249–6262.

(5) Mason, E. A.; McDaniel, E. W. *Transport Properties of Ions in Gases*; Wiley-VCH, 1988.

(6) Kanu, A. B.; Gribb, M. M.; Hill, H. H. Predicting optimal resolving power for ambient pressure ion mobility spectrometry. *Anal. Chem.* **2008**, *80*, 6610–6619.

(7) Kirk, A. T.; Grube, D.; Kobelt, T.; Wendt, C.; Zimmermann, S. High-Resolution High Kinetic Energy Ion Mobility Spectrometer Based on a Low-Discrimination Tristate Ion Shutter. *Anal. Chem.* **2018**, *90*, 5603–5611.

(8) Christopher R. M. Ryan; Alexander Haack; W. Scott Hopkins. Predicting Ion-Solvent Clustering in Differential Mobility Spectrometry using Anharmonic Thermochemistry. *ChemRxiv* **2023**.

(9) Schaefer, C.; Lippmann, M.; Beukers, M.; Beijer, N.; van de Kamp, B.; Knotter, J.; Zimmermann, S. Detection of Triacetone Triperoxide by High Kinetic Energy Ion Mobility Spectrometry. *Anal. Chem.* **2023**, *95*, 17099–17107.
